# Supplementary material for: Effects of low-temperature stress at different growth stages on rice physiology, pollen viability and yield in China’s cold region
Source: PLoS One. 2025 Aug 13;20(8):e0329441. doi: 10.1371/journal.pone.0329441 (PMC12349717; doi:10.1371/journal.pone.0329441)
Supplement: S1 File — S2 Fig. Effects of LTS on Pn. (a)-(c) the variation trend of the tillering, booting and heading stages. CK, under natural conditions (control); T1, under LTS of 17.5°C; T2, under LTS of 13.5°C; T3, under LTS of 11.5°C. 3d, 7d and 10d were duration of LTS. Error bars represented Mean ± SE (n = 3). Different letters in lowercase indicated significant difference of the data in all treatments at P < 0.05. S3 Fig. Effects of LTS on Ci. (a)-(c) the variation trend of the tillering, booting and heading stages. CK, under natural conditions (control); T1, under LTS of 17.5°C; T2, under LTS of 13.5°C; T3, under LTS of 11.5°C. 3d, 7d and 10d were duration of LTS. Error bars represented Mean ± SE (n = 3). Different letters in lowercase indicated significant difference of the data in all treatments at P < 0.05. S4 Fig. Effects of LTS on Gs. (a)-(c) the variation trend of the tillering, booting and heading stages. CK, under natural conditions (control); T1, under LTS of 17.5°C; T2, under LTS of 13.5°C; T3, under LTS of 11.5°C. 3d, 7d and 10d were duration of LTS. Error bars represented Mean ± SE (n = 3). Different letters in lowercase indicated significant difference of the data in all treatments at P < 0.05. S5 Fig. Effects of LTS on Tr. (a)-(c) the variation trend of the tillering, booting and heading stages. CK, under natural conditions (control); T1, under LTS of 17.5°C; T2, under LTS of 13.5°C; T3, under LTS of 11.5°C. 3d, 7d and 10d were duration of LTS. Error bars represented Mean ± SE (n = 3). Different letters in lowercase indicated significant difference of the data in all treatments at P < 0.05. S6 Fig. Effects of LTS on MDA. (a)-(c) the variation trend of the tillering, booting and heading stages. CK, under natural conditions (control); T1, under LTS of 17.5°C; T2, under LTS of 13.5°C; T3, under LTS of 11.5°C. 3d, 7d and 10d were duration of LTS. Error bars represented Mean ± SE (n = 3). Different letters in lowercase indicated significant difference of the data in al [file pone.0329441.s001.pdf]

Supporting information

S1 Fig. The hourly temperatures in the phytotron.

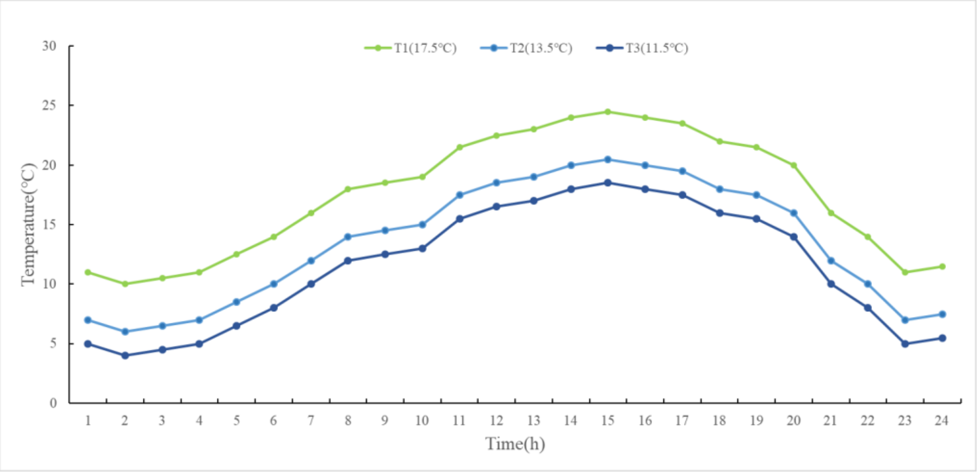

S1_file

Hour	T1(17.5℃)	T2(13.5℃)	T3(11.5℃)
0	11	7	5
1	10	6	4
2	10.5	6.5	4.5
3	11	7	5
4	12.5	8.5	6.5
5	14	10	8
6	16	12	10
7	18	14	12
8	18.5	14.5	12.5
9	19	15	13
10	21.5	17.5	15.5
11	22.5	18.5	16.5
12	23	19	17
13	24	20	18
14	24.5	20.5	18.5
15	24	20	18
16	23.5	19.5	17.5
17	22	18	16
18	21.5	17.5	15.5
19	20	16	14
20	16	12	10
21	14	10	8
22	11	7	5
23	11.5	7.5	5.5

S2 Fig. Effects of LTS on Pn. (a)-(c) the variation trend of the tillering, booting and heading stages. CK, under natural conditions (control); T1, under LTS of 17.5 °C; T2, under LTS of 13.5 °C; T3, under LTS of 11.5 °C. 3d, 7d and 10d were duration of LTS. Error bars represented Mean ± SE (n=3). Different letters in lowercase indicated significant difference of the data in all treatments at P<0.05.

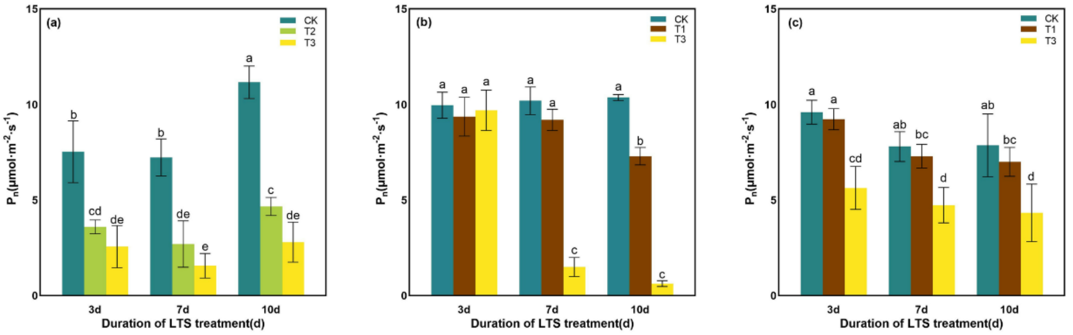

S2_file

tillering stage	duration of treatment(d)	CK			T2			T3		
	3d	5.8	7.8	9	3.3	3.5	4	1.5	2.5	3.7
	7d	6.4	7	8.3	1.6	2.5	4	1.1	1.3	2.3
	10d	10.2	11.5	11.8	4.3	4.5	5.2	1.6	3.3	3.5
booting stage	duration of treatment(d)	CK			T1			T2		
	3d	9.2	10.2	10.5	8.2	9.9	10	8.6	9.8	10.7
	7d	9.4	10.4	10.8	8.7	9.1	9.8	1	2	1.5
	10d	10.2	10.4	10.5	6.8	7.4	7.7	0.5	0.8	0.6
heading stage	duration of treatment(d)	CK			T1			T2		
	3d	8.9	9.8	10.1	8.7	9.2	9.8	4.4	5.9	6.6
	7d	7.3	7.4	8.7	6.6	7.5	7.8	3.7	5	5.5
	10d	6	8.5	9.1	6.2	7.1	7.7	2.6	5.1	5.3

S3 Fig. Effects of LTS on C_i. (a)-(c) the variation trend of the tillering, booting and heading stages. CK, under natural conditions (control); T1, under LTS of 17.5 °C; T2, under LTS of 13.5 °C; T3, under LTS of 11.5 °C. 3d, 7d and 10d were duration of LTS. Error bars represented Mean ± SE (n=3). Different letters in lowercase indicated significant difference of the data in all treatments at *P*<0.05.

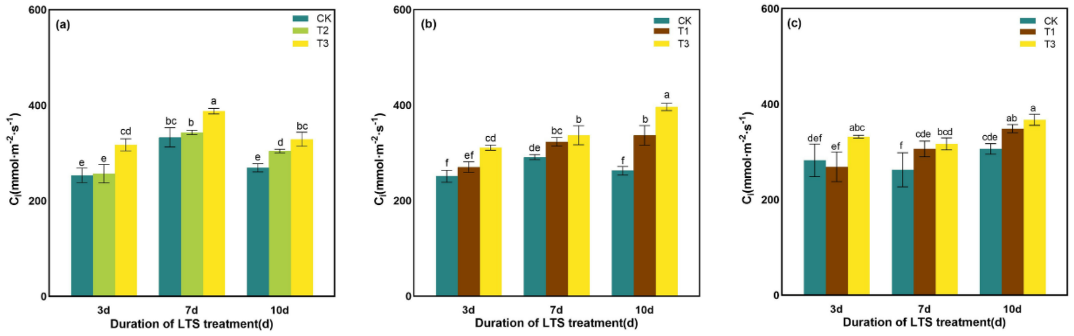

S3_file

tillering stage	duration of treatment(d)	CK			T2			T3		
	3d	236	259	265	243	249	279	309	311	332
	7d	318	325	356	338	346	346	381	390	392
	10d	261	269	278	300	306	307	313	332	342
booting stage	duration of treatment(d)	CK			T1			T2		
	3d	239	250	264	262	266	283	305	313	315
	7d	287	290	297	333	315	322	332	320	359
	10d	253	264	271	337	357	316	391	393	405
heading stage	duration of treatment(d)	CK			T1			T2		
	3d	243	298	306	273	236	298	329	332	335
	7d	277	222	289	325	295	299	304	318	329
	10d	296	305	318	352	355	339	378	369	355

S4 Fig. Effects of LTS on G_s. (a)-(c) the variation trend of the tillering, booting and heading stages. CK, under natural conditions (control); T1, under LTS of 17.5 °C; T2, under LTS of 13.5 °C; T3, under LTS of 11.5 °C. 3d, 7d and 10d were duration of LTS. Error bars represented Mean ± SE (n=3). Different letters in lowercase indicated significant difference of the data in all treatments at *P*<0.05.

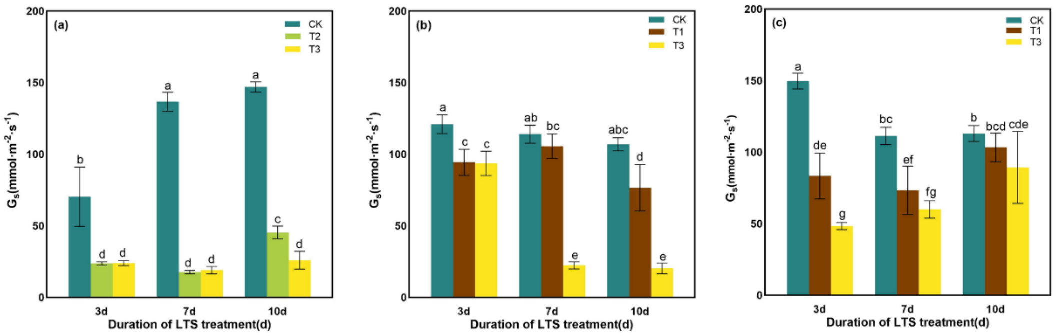

S4_file

tillering stage	duration of treatment(d)	CK			T2			T3		
	3d	48	74	89	23	23	25	23	23	26
	7d	131	135	144	17	17	19	17	18	22
	10d	143	148	150	41	45	50	21	24	33
booting stage	duration of treatment(d)	CK			T1			T2		
	3d	114	122	127	86	93	104	84	97	100
	7d	107	116	119	96	109	112	20	22	25
	10d	103	106	112	58	85	87	16	22	23
heading stage	duration of treatment(d)	CK			T1			T2		
	3d	144	150	155	79	70	101	46	51	48
	7d	105	112	117	69	59	92	56	57	67
	10d	108	112	119	93	104	113	61	109	98

S5 Fig. Effects of LTS on T_r. (a)-(c) the variation trend of the tillering, booting and heading stages. CK, under natural conditions (control); T1, under LTS of 17.5 °C; T2, under LTS of 13.5 °C; T3, under LTS of 11.5 °C. 3d, 7d and 10d were duration of LTS. Error bars represented Mean ± SE (n=3). Different letters in lowercase indicated significant difference of the data in all treatments at *P*<0.05.

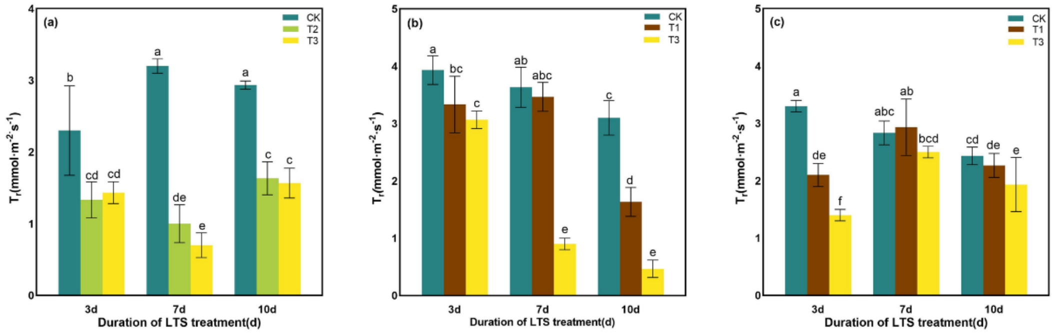

S5_file

tillering stage	duration of treatment(d)	CK			T2			T3		
	3d	1.6	2.5	2.8	1.3	1.6	1.1	1.3	1.4	1.6
	7d	3.1	3.2	3.3	1.3	0.8	0.9	0.6	0.6	0.9
	10d	2.9	2.9	3	1.5	1.5	1.9	1.4	1.5	1.8
booting stage	duration of treatment(d)	CK			T1			T2		
	3d	3.7	3.9	4.2	3	3.1	3.9	2.9	3.1	3.2
	7d	3.3	3.6	4	3.2	3.5	3.7	0.8	0.9	1
	10d	2.8	3.1	3.4	1.4	1.6	1.9	0.5	0.3	0.6
heading stage	duration of treatment(d)	CK			T1			T2		
	3d	3.2	3.3	3.4	2.3	1.9	2.1	1.3	1.4	1.5
	7d	2.6	2.9	3	2.7	2.6	3.5	2.5	2.4	2.6
	10d	2.3	2.4	2.6	2.2	2.5	2.1	1.4	2.1	2.3

S6 Fig. Effects of LTS on MDA. (a)-(c) the variation trend of the tillering, booting and heading stages. CK, under natural conditions (control); T1, under LTS of 17.5 °C; T2, under LTS of 13.5 °C; T3, under LTS of 11.5 °C. 3d, 7d and 10d were duration of LTS. Error bars represented Mean ± SE (n=3). Different letters in lowercase indicated significant difference of the data in all treatments at P<0.05.

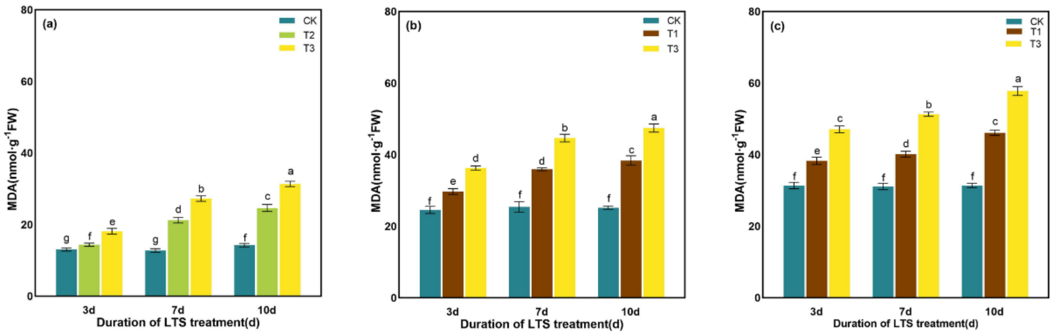

S6_file

tillering stage	duration of treatment(d)	CK			T2			T3		
	3d	12.71	13.56	12.89	13.9	14.76	14.47	17.79	19.07	17.55
	7d	12.19	12.89	13.19	21.03	22.06	20.55	27.18	26.56	28.06
	10d	14.78	13.78	14.22	24.33	25.73	23.85	31.44	30.55	32.07
booting stage	duration of treatment(d)	CK			T1			T2		
	3d	23.48	24.89	25.39	28.99	29.58	30.58	36.89	35.74	36.24
	7d	25.35	26.96	24.05	36.27	35.44	36.09	45.89	44.21	43.91
	10d	25.71	24.84	25.04	37.14	38.26	39.78	47.58	48.56	46.25
heading stage	duration of treatment(d)	CK			T1			T2		
	3d	30.44	31.43	32.24	38.83	37.11	38.9	46.2	48.09	46.97
	7d	30.2	31.91	31.18	39.21	40.32	40.84	51.24	50.76	51.93
	10d	31.9	31.54	30.74	45.53	46.97	45.84	56.43	58.74	58.21

S7 Fig. Effects of LTS on EL. (a)-(c) the variation trend of the tillering, booting and heading stages. CK, under natural conditions (control); T1, under LTS of 17.5 °C; T2, under LTS of 13.5 °C; T3, under LTS of 11.5 °C. 3d, 7d and 10d were duration of LTS. Error bars represented Mean ± SE (n=3). Different

letters in lowercase indicated significant difference of the data in all treatments at *P*<0.05.

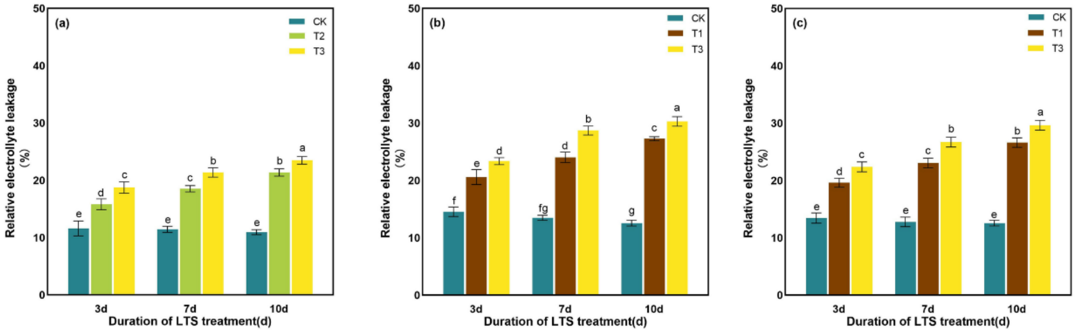

S7_file

tillering stage	duration of treatment(d)	CK			T2			T3		
	3d	11.588	12.896	10.299	15.487	16.897	15.098	18.397	17.962	19.856
	7d	11.348	12.04	10.985	18.596	17.965	19.059	21.598	22.066	20.489
	10d	10.489	10.987	11.387	20.789	21.259	22.049	23.594	22.789	24.089
booting stage	duration of treatment(d)	CK			T1			T2		
	3d	15.498	14.087	14.029	21.962	20.489	19.357	23.396	22.758	23.964
	7d	13.845	12.998	13.589	24.87	24.185	23.087	27.892	28.789	29.456
	10d	12.745	11.985	12.963	27.674	26.985	27.198	29.374	30.941	30.591
heading stage	duration of treatment(d)	CK			T1			T2		
	3d	13.589	14.269	12.495	18.962	20.489	19.357	21.396	22.758	22.964
	7d	12.687	13.674	11.996	23.87	22.185	23.087	26.892	25.789	27.456
	10d	13.049	12.596	12.054	25.674	26.985	27.198	29.374	28.941	30.591

S8 Fig. Effects of LTS on the production rate of $O_2^{\cdot-}$. (a)-(c) the variation trend of the tillering, booting and heading stages. CK, under natural conditions (control); T1, under LTS of 17.5 °C; T2, under LTS of 13.5 °C; T3, under LTS of 11.5 °C. 3d, 7d and 10d were duration of LTS. Error bars represented Mean ± SE (n=3). Different letters in lowercase indicated significant difference of the data in all treatments at *P*<0.05.

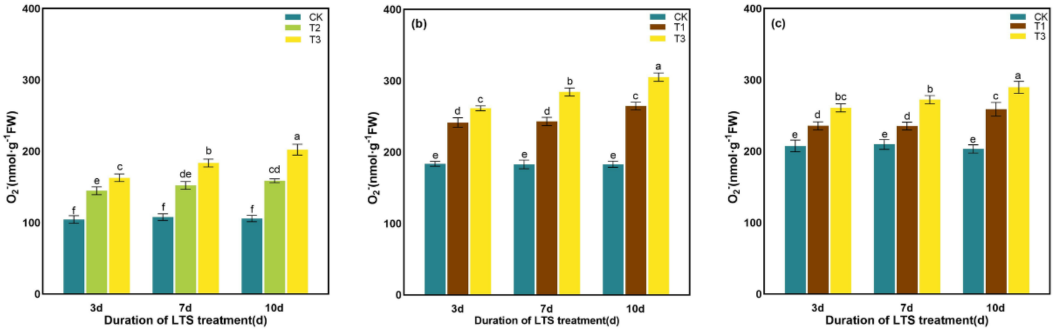

S8_file

tillering stage	duration of treatment(d)	CK			T2			T3		
	3d	100.9	110.59	101.86	138.59	145.97	149.36	160.9	158.79	168.99
	7d	102.5	110.97	109.88	148.5	149.6	158.4	182.6	178.7	189.57
	10d	104.9	110.59	101.96	158.74	155.96	161.3	200.9	210.6	195.49
booting stage	duration of treatment(d)	CK			T1			T2		
	3d	180.6	182.96	187.27	240.6	248.7	235.49	260.79	258.69	265.3
	7d	185.4	187.3	175.94	238.17	241.59	249.4	285.4	289.47	278.39
	10d	187.29	178.59	183.3	263.9	270.28	259.39	310.6	305.97	298.99
heading stage	duration of treatment(d)	CK			T1			T2		
	3d	209.59	214.6	198.57	235.9	241.4	230.25	260.47	266.95	255.49
	7d	210.6	215.96	202.46	241.3	234.59	230.57	278.69	267.46	271.39
	10d	201.6	198.65	209.79	258.96	268.48	249.29	298.48	289.12	281.6

Fig 9. Effects of LTS on H_2O_2. (a)-(c) the variation trend of the tillering, booting and heading stages.

CK, under natural conditions (control); T1, under LTS of 17.5 °C; T2, under LTS of 13.5 °C; T3, under LTS of 11.5 °C. 3d, 7d and 10d were duration of LTS. Error bars represented Mean ± SE (n=3). Different letters in lowercase indicated significant difference of the data in all treatments at *P*<0.05.

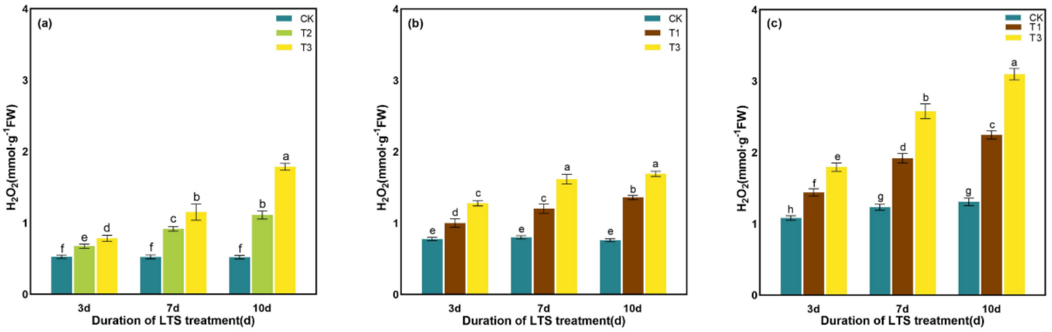

S9_file

tillering stage	duration of treatment(d)	CK			T2			T3		
	3d	0.4989	0.541	0.539	0.6404	0.6854	0.6974	0.7417	0.7854	0.8257
	7d	0.5269	0.5496	0.4929	0.8789	0.9278	0.9419	1.0357	1.1599	1.2599
	10d	0.5015	0.5479	0.506	1.0488	1.126	1.1599	1.7395	1.7827	1.8369
booting stage	duration of treatment(d)	CK			T1			T2		
	3d	0.7535	0.7863	0.7962	0.9417	1.0598	1.0029	1.2357	1.306	1.2896
	7d	0.7796	0.7936	0.8269	1.141	1.1987	1.2697	1.556	1.6871	1.605
	10d	0.741	0.7829	0.7594	1.3279	1.3599	1.3874	1.6546	1.6892	1.727
heading stage	duration of treatment(d)	CK			T1			T2		
	3d	1.0455	1.104	1.0964	1.3984	1.424	1.4964	1.7395	1.7857	1.857
	7d	1.1853	1.2699	1.2404	1.8499	1.9287	1.9823	2.4824	2.5689	2.6855
	10d	1.256	1.305	1.3599	2.1834	2.2579	2.2987	3.0077	3.126	3.1599

S10 Fig. Effects of LTS on SOD. (a)-(c) the variation trend of the tillering, booting and heading stages. CK, under natural conditions (control); T1, under LTS of 17.5 °C; T2, under LTS of 13.5 °C; T3, under LTS of 11.5 °C. 3d, 7d and 10d were duration of LTS. Error bars represented Mean ± SE (n=3). Different letters in lowercase indicated significant difference of the data in all treatments at *P*<0.05.

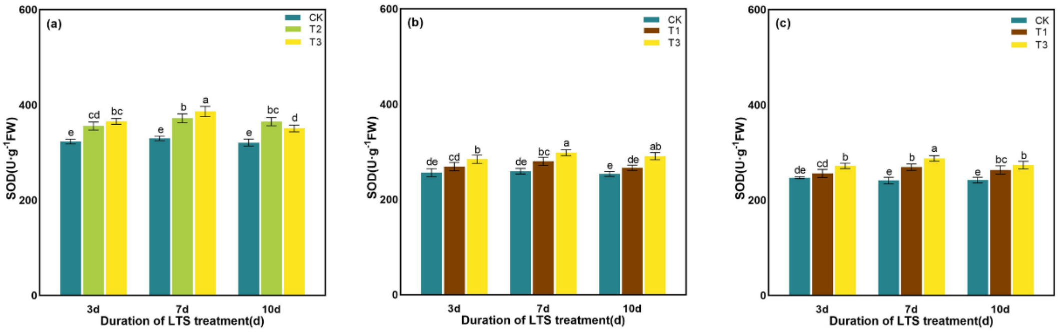

S10_file

tillering stage	duration of treatment(d)	CK			T2			T3		
	3d	321.67	319.09	328.61	349.46	352.04	365.46	358.34	367.92	369.63
	7d	333.6	331.1	324.6	372.04	362.86	381.41	374.45	388.44	395.65
	10d	328.69	314.06	320.06	362.87	357.43	374.05	345.21	358.69	347.86
booting stage	duration of treatment(d)	CK			T1			T2		
	3d	265.66	253.51	249.17	268.68	260.1	277.88	276.87	293.9	282.34
	7d	253.19	259.47	265.02	283.17	286.29	270.2	292.19	298.67	304.43
	10d	251.35	249.56	259.41	265.44	272.44	261.46	296.87	293.9	282.34
heading stage	duration of treatment(d)	CK			T1			T2		
	3d	247.15	244.97	249.24	265.66	253.51	249.17	268.25	269.24	278.25
	7d	243.94	233.17	246.29	262.1	275.2	270.56	286.87	293.9	282.34
	10d	248.65	240.29	237.61	265.59	271.01	253.64	265.31	281.47	274.02

S11 Fig. Effects of LTS on POD. (a)-(c) the variation trend of the tillering, booting and heading stages.

CK, under natural conditions (control); T1, under LTS of 17.5 °C; T2, under LTS of 13.5 °C; T3, under LTS of 11.5 °C. 3d, 7d and 10d were duration of LTS. Error bars represented Mean ± SE (n=3). Different letters in lowercase indicated significant difference of the data in all treatments at *P*<0.05.

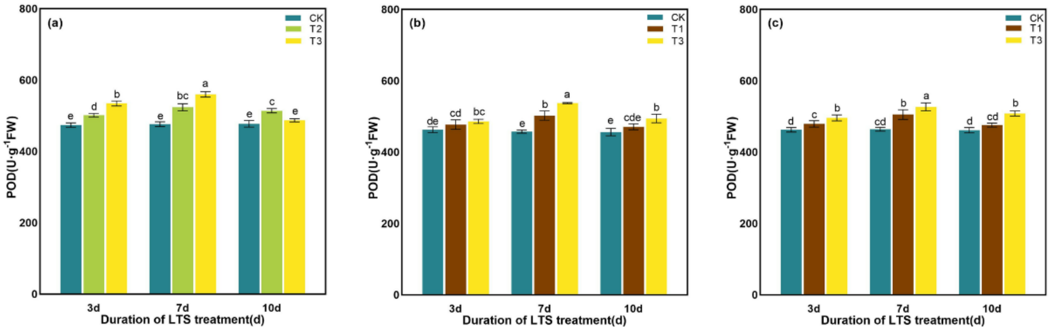

S11_file

tillering stage	duration of treatment(d)	CK			T2			T3		
	3d	467.6	479.65	474.83	501.57	496.51	506.63	542.04	528.31	533.37
	7d	469.05	479.89	480.61	534.81	521.08	515.3	568.32	553.87	557.48
	10d	471.69	488.19	472.53	508.8	513.13	520.36	484.95	492.17	484.22
booting stage	duration of treatment(d)	CK			T1			T2		
	3d	465.27	454.25	469.46	473.1	466.83	492.04	479.67	491.19	487.93
	7d	462.82	455.38	455.16	498.85	491.64	516.6	536.24	537.23	539.2
	10d	445.1	457.8	465.77	472.75	462.22	478.67	505.23	495.77	481.68
heading stage	duration of treatment(d)	CK			T1			T2		
	3d	469.5	460.69	457.79	480.12	469.85	487.58	486.95	496.95	504.05
	7d	463.05	459.5	470.15	507.17	517.9	491.17	539.75	521.22	519.67
	10d	468.97	454.24	461.87	470.02	481.26	476.53	505	516.55	503.84

S12 Fig. Effects of LTS on CAT. (a)-(c) the variation trend of the tillering, booting and heading stages. CK, under natural conditions (control); T1, under LTS of 17.5 °C; T2, under LTS of 13.5 °C; T3, under LTS of 11.5 °C. 3d, 7d and 10d were duration of LTS. Error bars represented Mean ± SE (n=3). Different letters in lowercase indicated significant difference of the data in all treatments at *P*<0.05.

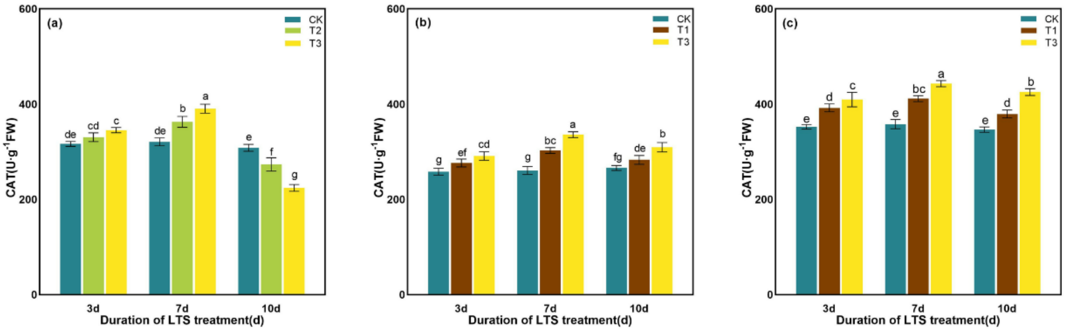

S12_file

tillering stage	duration of treatment(d)	CK			T2			T3		
	3d	317.94	320.85	310.89	332.58	338.59	320.75	346.61	350.12	339.56
	7d	321.13	312.39	328.96	361.7	351.96	374.96	391.05	380.96	399.74
	10d	308.94	300.96	315.39	276.59	285.48	258.14	224.67	216.85	230.96
booting stage	duration of treatment(d)	CK			T1			T2		
	3d	260.16	250.18	264.19	274.39	285.37	269.35	292.68	299.32	281.37
	7d	266.66	251.39	264.4	300.96	309.52	298.13	329.02	340.3	338.52
	10d	267.48	260.27	270.4	286.35	272.39	290.19	310.6	318.94	299.4
heading stage	duration of treatment(d)	CK			T1			T2		
	3d	357.72	348.12	351.85	382.76	395.37	398.36	404.47	426.52	397.63
	7d	368.78	350.23	354.86	410.36	418.35	405.96	450.36	438.35	440.4
	10d	347.15	340.75	351.96	387.15	380.46	370.7	432.44	425.38	418.37

S13 Fig. Effects of LTS on proline. (a)-(c) the variation trend of the tillering, booting and heading stages.

CK, under natural conditions (control); T1, under LTS of 17.5 °C; T2, under LTS of 13.5 °C; T3, under LTS of 11.5 °C. 3d, 7d and 10d were duration of LTS. Error bars represented Mean ± SE (n=3). Different letters in lowercase indicated significant difference of the data in all treatments at *P*<0.05.

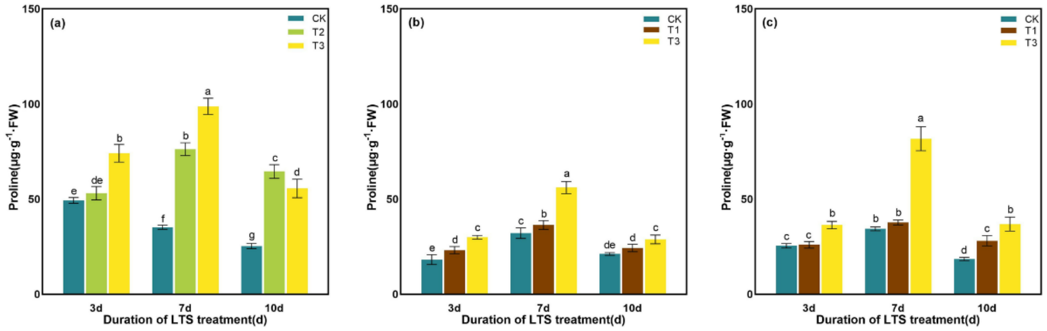

S13_file

tillering stage	duration of treatment(d)	CK			T2			T3		
	3d	49.301	47.771	50.831	53.107	49.597	56.617	74.118	69.438	78.798
	7d	35.239	34.109	36.369	76.268	72.958	79.578	98.768	94.508	103.03
	10d	25.313	23.953	26.673	64.522	60.952	68.092	55.588	50.698	60.478
booting stage	duration of treatment(d)	CK			T1			T2		
	3d	18.199	15.589	20.809	23.162	21.302	25.022	29.945	29.015	30.875
	7d	32.096	29.286	34.906	36.397	34.087	38.707	56.085	52.875	59.295
	10d	21.176	20.506	21.846	24.32	22.41	26.23	28.787	26.507	31.067
heading stage	duration of treatment(d)	CK			T1			T2		
	3d	25.478	24.308	26.648	25.974	24.234	27.714	36.397	34.467	38.327
	7d	34.412	33.392	35.432	37.721	36.441	39.001	81.765	75.495	88.035
	10d	18.529	17.659	19.399	28.125	25.415	30.835	36.893	33.223	40.563

S14 Fig. Effects of LTS on the soluble sugar. (a)-(c) the variation trend of the tillering, booting and heading stages. CK, under natural conditions (control); T1, under LTS of 17.5 °C; T2, under LTS of 13.5 °C; T3, under LTS of 11.5 °C. 3d, 7d and 10d were duration of LTS. Error bars represented Mean ± SE (n=3). Different letters in lowercase indicated significant difference of the data in all treatments at *P*<0.05.

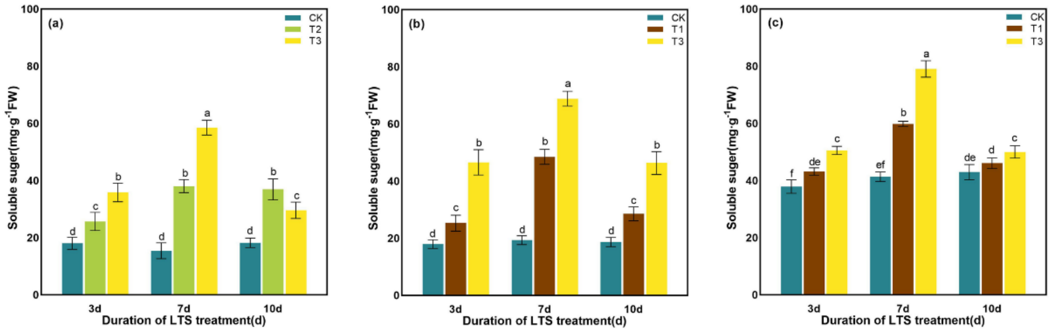

S14_file

tillering stage	duration of treatment(d)	CK			T2			T3		
	3d	15.968	17.895	20.198	25.952	28.695	22.395	32.195	36.851	38.486
	7d	15.966	12.396	17.862	37.925	35.841	40.395	55.699	58.963	60.862
	10d	18.529	19.659	16.399	36.893	33.223	40.563	28.525	27.415	32.835
booting stage	duration of treatment(d)	CK			T1			T2		
	3d	16.589	17.596	19.592	22.199	25.962	27.695	46.853	41.963	50.851
	7d	19.301	17.771	20.831	48.963	45.695	50.879	70.952	65.963	69.659
	10d	19.894	16.859	19.199	28.87	30.782	25.963	50.752	45.285	42.963
heading stage	duration of treatment(d)	CK			T1			T2		
	3d	37.251	35.895	40.469	42.742	42.099	44.589	50.871	51.76	48.967
	7d	39.598	42.957	41.395	58.973	59.845	60.752	79.684	75.915	81.597
	10d	41.915	40.941	45.952	46.852	43.951	47.395	51.955	47.682	50.395

S15 Fig. Effects of LTS on the soluble protein. (a)-(c) the variation trend of the tillering, booting and heading stages. CK, under natural conditions (control); T1, under LTS of 17.5 °C; T2, under LTS of 13.5 °C; T3, under LTS of 11.5 °C. 3d, 7d and 10d were duration of LTS. Error bars represented Mean ± SE (n=3). Different letters in lowercase indicated significant difference of the data in all treatments at *P*<0.05.

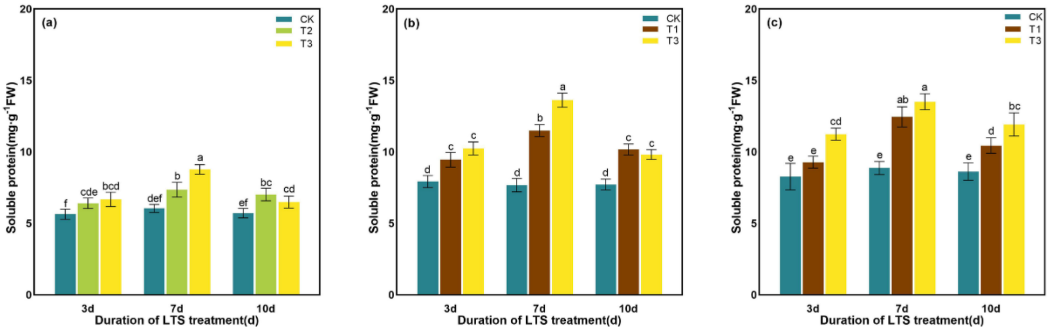

S15_file

tillering stage	duration of treatment(d)	CK			T2			T3		
	3d	5.3518	6.0307	5.5157	6.6888	6.5279	5.996	6.5739	6.2315	7.2185
	7d	6.2371	6.1652	5.7182	6.9164	7.2211	7.9227	9.1004	8.7825	8.428
	10d	5.6307	6.0752	5.4242	7.1723	6.5159	7.3624	6.0292	6.843	6.5868
**booting stage**	**duration of treatment(d)**	**CK**			**T1**			**T2**		
	3d	7.4641	8.2955	8.0095	8.8557	9.6888	9.802	10.271	10.681	9.7715
	7d	7.178	7.7228	8.1159	11.338	11.963	11.166	13.178	13.512	14.163
	10d	7.8469	7.2806	8.01	9.725	10.35	10.427	9.5915	10.216	9.6459
**heading stage**	**duration of treatment(d)**	**CK**			**T1**			**T2**		
	3d	9.2554	7.3982	8.1511	9.5442	9.4961	8.7977	11.29	11.637	10.79
	7d	8.3567	9.0278	9.2279	12.512	13.124	11.719	13.337	13.069	14.133
	10d	8.8483	9.0909	7.9373	10.063	11.066	10.203	11.048	12.652	12.063

S16 Fig. Effects of LTS on pollen viability. (a)-(c) the variation trend of the tillering, booting and heading stages. CK, under natural conditions (control); T1, under LTS of 17.5 °C; T2, under LTS of 13.5 °C; T3, under LTS of 11.5 °C. 3d, 7d and 10d were duration of LTS. Error bars represented Mean ± SE (n=3). Different letters in lowercase indicated significant difference of the data in all treatments at *P*<0.05.

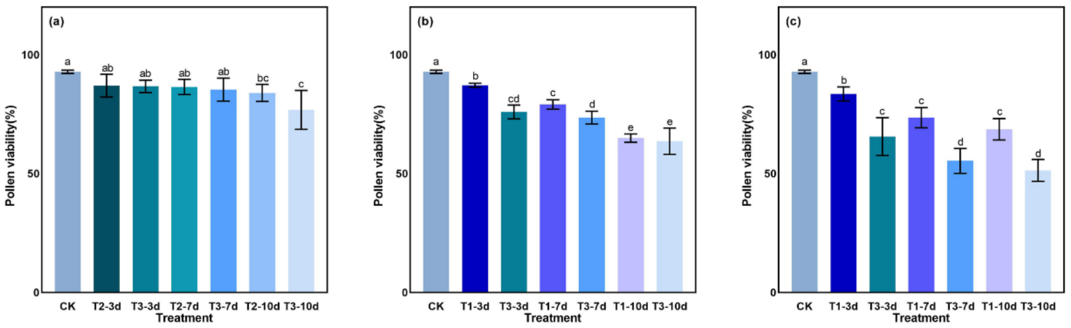

S16_file

tillering stage	treatment	CK	T2-3d	T3-3d	T2-7d	T3-7d	T2-10d	T3-10d
	Sample 1	92.222	91.919	86.638	82.759	90.476	80	77.5
	Sample 2	92.683	82.353	89.316	88.136	80.899	84.706	84.615
	Sample 3	93.59	86.667	84.071	88.462	84.615	87.059	68.293
booting stage	treatment	CK	T1-3d	T3-3d	T1-7d	T3-7d	T1-10d	T3-10d
	Sample 1	92.222	88.06	72.727	79.07	76.608	63.158	63.158
	Sample 2	92.683	86.42	78.431	77.108	71.598	65	58.333
	Sample 3	93.59	86.957	76.596	81.053	72.414	66.667	69.388
heading stage	treatment	CK	T1-3d	T3-3d	T1-7d	T3-7d	T1-10d	T3-10d
	Sample 1	92.222	83.019	71.875	68.75	50	71.951	47.619
	Sample 2	92.683	86.667	56.627	75	60.465	63.514	50
	Sample 3	93.59	80.851	68.182	76.829	55.556	70.476	56.471

S17 Fig. The Pearson correlation matrix between Pn, MDA as Well as Yield and Yield Related Parameters under LTS at tillering (T), booting (B), and heading (H) stages. *, ** and *** represent the significant correlation at $p<0.05$, $p<0.01$ and $p<0.001$, respectively.

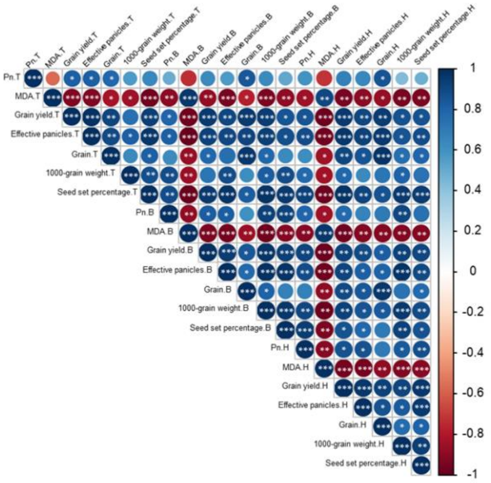

S17_file

tillering stage	group	Pn	MDA	Grain yield	Effective panicles	Grain	1000-grainweight	Seed set percentage
	CK	8.64	13.36	37.47	17.66666667	170.66667	22.80555556	89.97477198
	T3-3d	2.567	18.14	23.761	13.66666667	153.66667	22.61111111	81.16172026
	T3-7d	1.567	27.27	19.55866667	12	149.33333	22.25	76.28991121
	T3-10d	2.8	31.35	18.026	11.33333333	144	21.78333333	70.96861472
	T2-3d	3.6	14.38	29.36766667	15.66666667	155	22.65555556	87.94910865
	T2-7d	2.7	21.21	27.888	14	151.33333	22.46666667	83.25028412
	T2-10d	4.667	24.64	23.274	13.66666667	145.66667	22.6978979	80.044504
booting stage	CK	10.178	25.08	37.47	17.66666667	170.66667	22.80555556	89.97477198
	T3-3d	9.7	36.29	14.95466667	14.33333333	106.33333	21.76333333	64.29143366
	T3-7d	1.5	44.67	8.570666667	10.33333333	103.53333	20.75555556	40.48482705
	T3-10d	0.633	47.46	4.21	7.666666667	90.633333	20.51065666	21.90434582
	T1-3d	9.367	29.72	31.67133333	16.33333333	120.06667	22.42222222	93.57648549
	T1-7d	9.2	35.933	30.454	16	119.23333	22.27666667	85.77838494
	T1-10d	7.3	38.39	15.25	13	100.36667	21.51333333	70.93529845
heading stage	CK	8.42	31.29	37.47	17.66666667	170.66667	22.80555556	89.97477198
	T3-3d	5.633	47.09	21.72	13	127.76667	22.31111111	67.9848726
	T3-7d	4.733	51.31	18.19033333	12.33333333	111.4	21.52435334	63.19378042
	T3-10d	4.333	57.79	9.429	11.33333333	103.23333	20.80709294	44.87705166
	T1-3d	9.233	38.28	31.964	17.33333333	133.93333	22.76666667	94.17195688
	T1-7d	7.3	40.12	23.825	13.66666667	121.03333	22.6	73.67600053
	T1-10d	7	46.11	17.606	12	116.9	21.6	57.81377851

S18 Fig. The effect of LTS on chloroplast ultrastructure of rice at booting stage. Chloroplast structure and thylakoid organization in control and LTS were analyzed by transmission electron

microscopy (TEM). CK, under the natural conditions (control) for duration of 3, 7 and 10 days (A, B and C); T1, LTS of mean temperature 17.5°C for duration of 3, 7 and 10 days (D, E and F); T3, LTS of mean temperature 11.5°C for duration of 3, 7 and 10 days (G, H and I). G: granum; SL: stroma lamellae; GL: grana lamellae; SG: starch grain; OG: osmophilic gramules; CHM: chloroplast membrane. Bar = 500 nm.

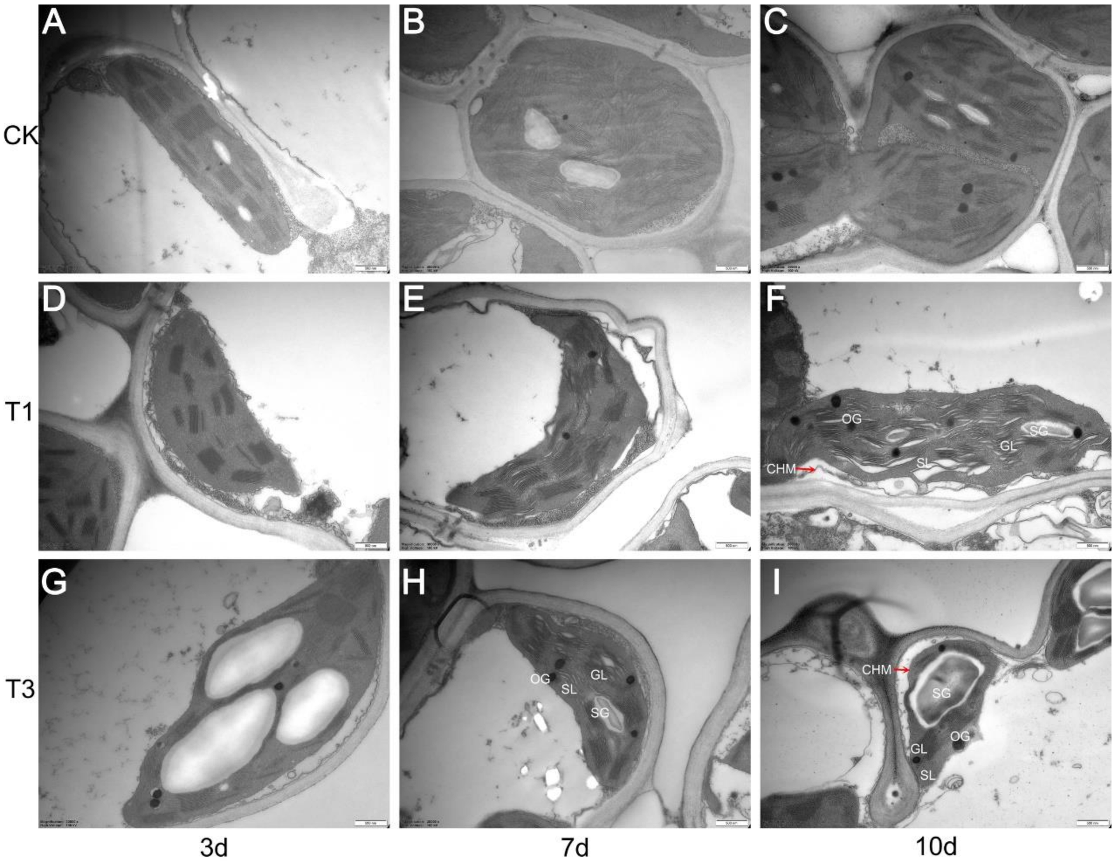

S19 Fig. The Pearson correlation matrix between Pollen Viability as Well as Yield and Yield Related Parameters under LTS. *, ** and *** represent the significant correlation at p<0.05, p<0.01 and p<0.001, respectively.

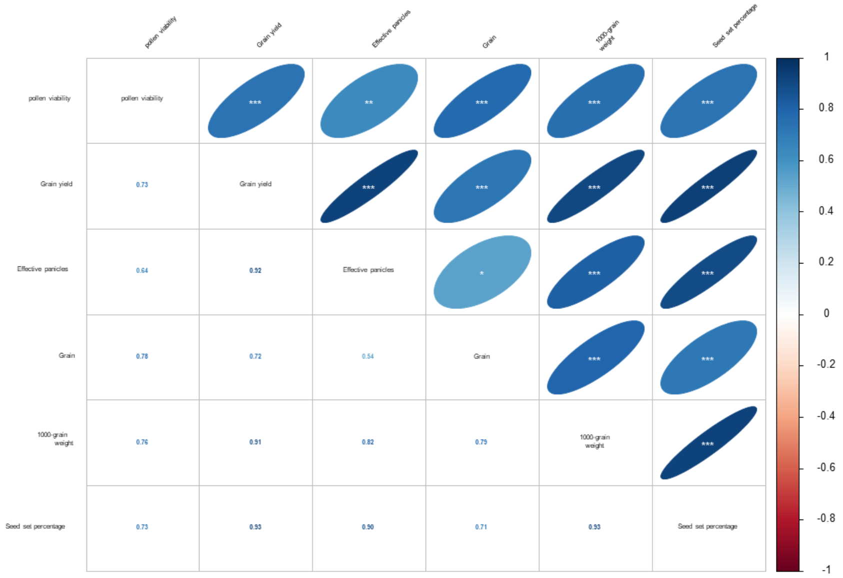

S19_file

group	pollen viability	Grain yield	Effective panicles	Grain	1000-grainweight	Seed set percentage
CK	92.83	37.47	17.66666667	170.6666667	22.80555556	89.97477198
T3-3d	86.67498894	23.761	13.66666667	153.6666667	22.61111111	81.16172026
T3-7d	85.3301505	19.55866667	12	149.3333333	22.25	76.28991121
T3-10d	76.80268918	18.026	11.33333333	144	21.78333333	70.96861472
T2-3d	86.97959992	29.36766667	15.66666667	155	22.65555556	87.94910865
T2-7d	86.45191746	27.888	14	151.3333333	22.46666667	83.25028412
T2-10d	83.92156863	23.274	13.66666667	145.6666667	22.6978979	80.044504
T3-3d	75.91812999	14.95466667	14.33333333	106.3333333	21.76333333	64.29143366
T3-7d	73.53987112	8.570666667	10.33333333	103.5333333	20.75555556	40.48482705
T3-10d	63.62632772	4.21	7.666666667	90.63333333	20.51065666	21.90434582
T1-3d	87.14532544	31.67133333	16.33333333	120.0666667	22.42222222	93.57648549
T1-7d	79.07694425	30.454	16	119.2333333	22.27666667	85.77838494
T1-10d	64.94152047	15.25	13	100.3666667	21.51333333	70.93529845
T3-3d	65.56110807	21.72	13	127.7666667	22.31111111	67.9848726
T3-7d	55.34022395	18.19033333	12.33333333	111.4	21.52435334	63.19378042
T3-10d	51.36321195	9.429	11.33333333	103.2333333	20.80709294	44.87705166
T1-3d	83.51219947	31.964	17.33333333	133.9333333	22.76666667	94.17195688
T1-7d	73.52642276	23.825	13.66666667	121.0333333	22.6	73.67600053
T1-10d	68.6469745	17.606	12	116.9	21.6	57.81377851

S1 Table. The average daily mean temperature, average daily minimum temperature, and average daily maximum temperature of each CK group of cultivated rice grown under natural conditions in 2023 and 2024.

Growth stage	Duration of LTS treatment	Average daily mean temperature(°C) (CK)	Average daily minimum temperature(°C)	Average daily maximum temperature(°C)
Tillering stage	D3	18.2	15.1	21.2
	D7	20.4	15.8	24.8
	D10	21.2	16.9	25.7
Booting stage	D3	19.9	16.0	24.2
	D7	23.3	18.4	28.2
	D10	23.6	19.0	28.1
Heading stage	D3	24.3	19.5	29.0
	D7	24.7	20.3	29.4
	D10	23.9	20.4	28.1

S2 Table. Effect of LTS on grain yield and its components at different growth stages.

Growth stages	Treatment	Treatment days/d	Effective panicles (number/pot)	Grain/panicle	Seed set percentage (%)	1000-grain weight (g)	Grain yield (g/pot)
Tillering stage	CK		17.67±2.52a	170.67±14.50a	89.97±1.46a	22.81±0.27a	37.47±6.60a
	T2	3	15.67±2.08a	155.00±12.77ab	87.95±1.61a	22.66±0.28a	29.37±0.85b

	T3		13.67±4.73a	153.67±7.02ab	81.16±1.77b	22.61±0.32a	23.76±1.22bcd
	T2	7	14.00±2.00a	151.33±9.02ab	83.25±2.40b	22.47±0.17ab	27.89±0.98bc
	T3		12.00±3.61a	149.33±13.05ab	76.29±2.31c	22.25±0.57ab	19.56±2.37d
	T2	10	13.67±0.58a	145.67±11.50ab	80.04±2.92b	22.70±0.52a	23.27±0.94cd
	T3		11.33±4.04a	144.00±15.52b	70.97±2.03d	21.78±0.37c	18.03±3.96d
Booting stage	CK		17.67±2.52a	170.67±14.50a	89.97±1.46ab	22.81±0.27a	37.47±6.60a
	T1	3	16.33±1.53a	120.07±5.42b	93.58±1.13a	22.42±0.34ab	31.67±2.74a
	T3		14.33±2.52ab	106.33±3.05bc	64.29±2.99d	21.76±0.58b	14.95±3.85b
	T1	7	16.00±3.61a	119.23±5.03b	85.78±2.42b	22.28±0.39ab	30.45±5.50a
	T3		10.33±1.53bc	103.53±1.79cd	40.48±3.41e	20.76±0.90cd	8.57±0.77bc
	T1	10	13.00±3.61ab	100.37±7.46cd	70.94±4.23c	21.51±0.48bc	15.25±3.50b
	T3		7.67±1.53c	90.63±10.02d	21.90±2.48f	20.51±0.60d	4.21±1.91c
Heading stage	CK		17.67±2.52a	170.67±14.50a	89.97±1.46a	22.81±0.27a	37.47±6.60a
	T1	3	17.33±1.53ab	133.93±5.69b	94.17±1.17a	22.77±0.88a	31.96±6.38a
	T3		13.00±2.65c	127.77±5.49bc	67.98±3.63bc	22.31±1.16ab	21.72±5.12b
	T1	7	13.67±2.52bc	121.03±7.17bcd	73.68±2.12b	22.60±0.55a	23.83±1.25b
	T3		12.33±2.52c	111.40±8.41de	63.19±6.88cd	21.52±0.93ab	18.19±2.28b
	T1	10	12.00±1.00c	116.90±6.31cde	57.81±3.38d	21.60±0.58ab	17.61±2.10b
	T3		11.33±1.53c	103.23±9.23e	44.88±8.37e	20.81±1.08c	9.43±1.71c
