# Supplementary material for: Effects of low-temperature stress at different growth stages on rice physiology, pollen viability and yield in China’s cold region
Source: PLoS One. 2025 Aug 13;20(8):e0329441. doi: 10.1371/journal.pone.0329441 (PMC12349717; doi:10.1371/journal.pone.0329441)
Supplement: Attachment — Submitted filename: PONE-D-25-20271.pdf [file pone.0329441.s002.pdf]

PLOS ONE

Effects of Low-Temperature Stress at Different Growth Stages on Rice Physiology, Pollen Viability and Yield in China’s Cold Region

--Manuscript Draft--

**Manuscript Number:**	PONE-D-25-20271
**Article Type:**	Research Article
**Full Title:**	Effects of Low-Temperature Stress at Different Growth Stages on Rice Physiology, Pollen Viability and Yield in China’s Cold Region
**Short Title:**	Cold Stress Effects on Rice Physiology, Pollen, and Yield Across Growth Stages in China’s Cold Region
**Corresponding Author:**	Lixia Jiang<br>Heilongjiang Province Institute of Meteorological Science<br>harbin, Heilongjiang province CHINA
**Keywords:**	low-temperature stress; rice; physiology; yield; growth stage
**Abstract:**	LTS constitutes a primary limiting factor for rice production in high-latitude regions. In this field experiment, we conducted a one-year temperature-controlled study to investigate the effects of low-temperatures and their durations at the tillering, booting, and heading stages on japonica rice (Longgeng 31). The results showed that the LTS-induced decrease in net photosynthetic rate was primarily attributed to stomatal limiting factor at the tillering stage but non-stomatal limiting factor at the booting and heading stages. The increasing intensity and prolonged duration of LTS resulted in distorted chloroplast morphology. The continuous low-temperature reduced plant height, and stress response to low-temperature in different stages was the same. The concentrations of the antioxidant and osmotic regulation systems in rice peaked after 7 days of LTS treatment, indicating that the stress response of rice to LTS had reached a critical threshold. The grain yield, effective panicles, grain number per panicle, 1000-grain weight and seed setting percentage of rice decreased the most at booting stage, followed by heading stage and tillering stage. LTS at the booting and heading stages could significantly reduce pollen viability, while LTS at the tillering stage had no significant effect on pollen viability.
**Order of Authors:**	Lifeng Guo, M.D.
	Xiaodong Du
	Jianing Chang
	Jingjin Gong
	Zheng Chu
	Jiajia Lv
	Lixia Jiang
**Additional Information:**	
**Question**	**Response**
**Financial Disclosure**<br><br>Enter a financial disclosure statement that describes the sources of funding for the work included in this submission. Review the submission guidelines for detailed requirements. View published research articles from *PLOS ONE* for specific examples.	Yes

Powered by Editorial Manager® and ProduXion Manager® from Aries Systems Corporation

This statement is required for submission and **will appear in the published article** if the submission is accepted. Please make sure it is accurate.<br><br>**Funded studies**<br>Enter a statement with the following details:<br>• Initials of the authors who received each award<br>• Grant numbers awarded to each author<br>• The full name of each funder<br>• URL of each funder website<br>• Did the sponsors or funders play any role in the study design, data collection and analysis, decision to publish, or preparation of the manuscript?<br><br>Did you receive funding for this work?	
Please add funding details.<br>as follow-up to "**Financial Disclosure**<br><br>Enter a financial disclosure statement that describes the sources of funding for the work included in this submission. Review the submission guidelines for detailed requirements. View published research articles from *PLOS ONE* for specific examples.<br><br>This statement is required for submission and **will appear in the published article** if the submission is accepted. Please make sure it is accurate.<br><br>**Funded studies**<br>Enter a statement with the following details:<br>• Initials of the authors who received each award<br>• Grant numbers awarded to each author<br>• The full name of each funder<br>• URL of each funder website<br>• Did the sponsors or funders play any role in the study design, data collection and analysis, decision to publish, or preparation of the manuscript?	the National Key Research and Development Program of China(No. 2022YFD2300201)<br>the Natural Science Foundation of Heilongjiang Province(No. LH2024D020)<br>the Joint Foundation on Regional Meteorological S & T Collaborative Innovation of Northeast China(No. 2024GX006)

Powered by Editorial Manager® and ProduXion Manager® from Aries Systems Corporation

Did you receive funding for this work?"	
Please select the country of your main research funder (please select carefully as in some cases this is used in fee calculation).<br>as follow-up to "**Financial Disclosure**<br><br>Enter a financial disclosure statement that describes the sources of funding for the work included in this submission. Review the submission guidelines for detailed requirements. View published research articles from *PLOS ONE* for specific examples.<br><br>This statement is required for submission and **will appear in the published article** if the submission is accepted. Please make sure it is accurate.<br><br>**Funded studies**<br>Enter a statement with the following details:<br>• Initials of the authors who received each award<br>• Grant numbers awarded to each author<br>• The full name of each funder<br>• URL of each funder website<br>• Did the sponsors or funders play any role in the study design, data collection and analysis, decision to publish, or preparation of the manuscript?<br><br>Did you receive funding for this work?"	CHINA - CN
**Competing Interests**<br><br>Use the instructions below to enter a competing interest statement for this submission. On behalf of all authors, disclose any competing interests that could be perceived to bias this work—acknowledging all financial support and any other relevant financial or non-financial competing interests.<br><br>This statement is required for submission and **will appear in the published article** if	The authors have declared that no competing interests exist.

the submission is accepted. Please make sure it is accurate and that any funding sources listed in your Funding Information later in the submission form are also declared in your Financial Disclosure statement.<br><br>View published research articles from *PLOS ONE* for specific examples.<br><br>**NO authors have competing interests**<br><br>Enter: *The authors have declared that no competing interests exist.*<br><br>**Authors with competing interests**<br><br>Enter competing interest details beginning with this statement:<br><br>*I have read the journal's policy and the authors of this manuscript have the following competing interests: [insert competing interests here]*<br><br>* typeset	
**Ethics Statement**<br><br>Enter an ethics statement for this submission. This statement is required if the study involved:<br><br>• Human participants<br>• Human specimens or tissue<br>• Vertebrate animals or cephalopods<br>• Vertebrate embryos or tissues<br>• Field research<br><br>Write "N/A" if the submission does not require an ethics statement.<br><br>General guidance is provided below. Consult the submission guidelines for detailed instructions. **Make sure that all information entered here is included in the Methods section of the manuscript.**	N/A

**Format for specific study types**<br><br>**Human Subject Research (involving human participants and/or tissue)**<br>• Give the name of the institutional review board or ethics committee that approved the study<br>• Include the approval number and/or a statement indicating approval of this research<br>• Indicate the form of consent obtained (written/oral) or the reason that consent was not obtained (e.g. the data were analyzed anonymously)<br><br>**Animal Research (involving vertebrate animals, embryos or tissues)**<br>• Provide the name of the Institutional Animal Care and Use Committee (IACUC) or other relevant ethics board that reviewed the study protocol, and indicate whether they approved this research or granted a formal waiver of ethical approval<br>• Include an approval number if one was obtained<br>• If the study involved *non-human primates*, add *additional details* about animal welfare and steps taken to ameliorate suffering<br>• If anesthesia, euthanasia, or any kind of animal sacrifice is part of the study, include briefly which substances and/or methods were applied<br><br>**Field Research**<br><br>Include the following details if this study involves the collection of plant, animal, or other materials from a natural setting:<br>• Field permit number<br>• Name of the institution or relevant body that granted permission	
**Data Availability**<br><br>Authors are required to make all data underlying the findings described fully available, without restriction, and from the time of publication. PLOS allows rare exceptions to address legal and ethical concerns. See the PLOS Data Policy and FAQ for detailed information.	Yes - all data are fully available without restriction

A Data Availability Statement describing where the data can be found is required at submission. Your answers to this question constitute the Data Availability Statement and **will be published in the article**, if accepted.<br><br>**Important:** Stating 'data available on request from the author' is not sufficient. If your data are only available upon request, select 'No' for the first question and explain your exceptional situation in the text box.<br><br>Do the authors confirm that all data underlying the findings described in their manuscript are fully available without restriction?	
**Describe where the data may be found in full sentences. If you are copying our sample text, replace any instances of XXX with the appropriate details.**<br><br>• If the data are **held or will be held in a public repository**, include URLs, accession numbers or DOIs. If this information will only be available after acceptance, indicate this by ticking the box below. For example: *All XXX files are available from the XXX database (accession number(s) XXX, XXX.*).<br>• If the data are all contained **within the manuscript and/or Supporting Information files**, enter the following: *All relevant data are within the manuscript and its Supporting Information files.*<br>• If neither of these applies but you are able to provide **details of access elsewhere**, with or without limitations, please do so. For example:<br><br>*Data cannot be shared publicly because of [XXX]. Data are available from the XXX Institutional Data Access / Ethics Committee (contact via XXX) for researchers who meet the criteria for access to confidential data.*<br><br>*The data underlying the results presented in the study are available from (include the name of the third party*	All relevant data are within the manuscript and its Supporting Information files.

and contact information or URL).<br>• This text is appropriate if the data are owned by a third party and authors do not have permission to share the data.<br><br>* typeset	
Additional data availability information:	Tick here if the URLs/accession numbers/DOIs will be available only after acceptance of the manuscript for publication so that we can ensure their inclusion before publication.

Manuscript Click here to access/download;Manuscript;manuscript.docx

Effects of Low-Temperature Stress at Different Growth Stages on Rice Physiology, Pollen Viability and Yield in China's Cold Region

Lifeng Guo[1,2], Xiaodong Du[3], Jianing Chang[4], Jingjin Gong[1,2], Zheng Chu[1,2], Jiajia Lv[1,2] and Lixia Jiang[1,2*]

[1] Heilongjiang Province Institute of Meteorological Science, Harbin 150030, China; kysglf@163.com (L.G.); gjj1949@163.com (J.G.); chuzheng1985@126.com (Z.C.); wflljj@163.com (J.L.)
[2] Wuying National Climatological Observatory, Harbin 150030, China;
[3] Rice Research Institute of Heilongjiang Academy of Agricultural Sciences, Jiamusi 154026, China; duxiaodong.com.cn@163.com (X.D.)
[4] School of Resources and Environment, Northeast Agricultural University, Harbin 150030, China; hljcjn0430@163.com (J.C.)
* Correspondence: hljjlx@163.com

Abstract: LTS constitutes a primary limiting factor for rice production in high-latitude regions. In this field experiment, we conducted a one-year temperature-controlled study to investigate the effects of low-temperatures and their durations at the tillering, booting, and heading stages on japonica rice (Longgeng 31). The results showed that the LTS-induced decrease in net photosynthetic rate was primarily attributed to stomatal limiting factor at the tillering stage but non-stomatal limiting factor at the booting and heading stages. The increasing intensity and prolonged duration of LTS resulted in distorted chloroplast morphology. The continuous low-temperature reduced plant height, and stress response to low-temperature in different stages was the same. The concentrations of the antioxidant and osmotic regulation systems in rice peaked after 7 days of LTS treatment, indicating that the stress response of rice to LTS had reached a critical threshold. The grain yield, effective panicles, grain number per panicle, 1000-grain weight and seed setting percentage of rice decreased the most at booting stage, followed by heading stage and tillering stage. LTS at the booting and heading stages could significantly reduce pollen viability, while LTS at the tillering stage had no significant effect on pollen viability.

Keywords: low-temperature stress; rice; physiology; yield; growth stage

1. Introduction

The rice plant (*Oryza sativa L.*) is a crucial staple crop for more than half of the global population, particularly in Asian nations[1, 2]. The consumption of rice in China is essential, as it serves as a primary food source for over 65% of the population[3], despite having less than half the global average of arable land per capita[4]. The cultivation of rice spans a wide range of climates, from tropical and subtropical to temperate and even cold regions, due to its preference for specific temperature conditions[5]. Its growth has certain requirements for meteorological conditions such as water and light, and it is particularly sensitive to temperature conditions[6, 7]. Low-temperature is one of the main limiting factors for rice production in high-latitude or high-altitude regions[8-10]. In recent years, under the background of global warming, the frequency of extreme weather events has changed, and the occurrence of periodic low-temperature may increase[11, 12]. As the world's largest rice producing country, China has suffered serious impact of low-temperature events[13, 14], especially as the high-latitude northeast China, the continuous low-temperature in the growing season has seriously affected

the growth and development of rice[15, 16]. Unlike other gramineous crops, rice is more vulnerable to low-temperature stress (LTS). ~~LTS in vegetative growth process can delay the growth stage~~[17, 18], ~~while in reproductive growth process, pollen sterility, empty shell rate increase and serious yield reduction can be caused~~[19]. The effect of LTS on rice production has always been the focus of rice meteorological research.

When the external environment changes, the plant's resistance to environmental stress is a physicochemical reaction process involving the coordination of multiple systems, which is not only affected by the genetic characteristics of the species, but also restricted by the environment. The LTS is a crucial abiotic stress in rice, exerting significant control over both rice production and geographical distribution[20, 21]. When exposed to low-temperatures, rice cells undergo a series of morphological, physiological, and biochemical changes in their structure as well as alterations in various cellular substances to sustain normal growth. Consequently, this phenomenon primary effect of low-temperature and cold injury on rice production is the reduction in yield, primarily attributed to the reduction of anther, the increase of sterile pollen in anther, and the decrease of seed setting rate, all of which collectively affect rice productivity[22]. The effect of low-temperature injury usually destroys energy source and metabolic balance of rice[23, 24], inhibits photosynthetic rate and reduces leaf area, thereby reducing plant biomass accumulation and yield[25].

The effect of LTS on physiology and biochemistry of rice is a complicated process. The stability and function of cell membrane, photosynthetic efficiency, antioxidant system and penetration regulation synergistic changes were observed in rice at low-temperatures[26, 27]. Antioxidant enzymes, including peroxidase (POD), superoxide dismutase (SOD), and catalase (CAT), act as protective enzyme systems to limit the rise of free radicals and prevent them from causing damage to rice cells, and maintain the balance between antioxidant system and free radicals[28]. LTS generates excessive amounts of free radicals, peroxides, and reactive oxygen species (ROS), thereby causing damage to the antioxidant system of rice. However, the defense mechanisms of antioxidant enzymes can effectively mitigate their harmful effects on rice[29]. The accumulation of proline (Pro) in response to LTS is an adaptive mechanism that enhances rice tolerance and can be expressed as a dynamic equilibrium between Pro accumulation and the threshold for LTS tolerance[30-32]. The accumulation of soluble proteins (SP) is also observed in response to LTS, as they possess osmotic adjustment functions and serve to protect rice cells from dehydration damage[33].

As is well known, LTS can induce excessive accumulation of ROS, thereby seriously damaging the cell membrane composition, chloroplast ultrastructure and plastid structure in rice plants[34], restricting photosynthesis and reducing substance production[35]. The photosynthetic parameters are commonly employed as indicators of the extent of environmental stress experienced by plants. The LTS can affect the intercellular carbon dioxide concentration and stomatal conductance in rice[36]. Stomata, as a crucial conduit for of carbon assimilation in plant ecosystem, is easily restricted by various environmental and physiological factors[37]. The opening and closing of stomata in leaves play a crucial role in regulating the intercellular carbon dioxide concentration, thereby affecting photosynthesis and the biochemical processes within chloroplasts[38]. The primary limitations on photosynthesis include both stomatal and non-stomatal factors. Stomatal limitation refers to the restriction of carbon dioxide supply diffusing through stomata into the intercellular spaces of leaf tissues. Non-stomatal limitation encompasses all processes that hinder the rate of photosynthesis in leaves, excluding those related to stomata. These include the diffusion of carbon dioxide from the intercellular spaces within leaf cells to the dark reaction sites in chloroplasts, as well as various other biochemical factors that impact photosynthetic rates[39, 40].

The growth of rice at each stage is dependent on a specific minimum optimum

temperature, and low-temperatures hinder the accumulation of dry matter[41]. Previous studies have shown that rice is highly sensitive to low-temperatures during its reproductive stage. Exposure to these conditions can result in reduced pollen viability, decreased effective tiller count, increased empty grain rate, lowered thousand-grain weight and ultimately lead to a decline in rice yield[42, 43]. The cold tolerance of various rice cultivars varies. The japonica rice cultivars originating from Korea and Japan generally demonstrate higher resistance to low-temperatures compared to those from China and the majority of Russia, for example[44, 45]. The tolerance of rice to low-temperatures is not only dependent on the source or variety, but also on the growth stage, as well as the intensity and duration of the LTS[46, 47]. However, the frequent occurrence of extreme weather events resulting from climate change has further exacerbated the effect of LTS on rice production[48]. The necessity to assess and quantify the effect of LTS on rice production arises from the projected climate changes in the future and their potential ramifications on agricultural yields.

Multiple studies have been conducted to identify the growth stages that exhibit the highest susceptibility to LTS, as well as to elucidate the underlying mechanisms that account for how LTS affects rice yields. The effect of LTS on rice yield can manifest through various mechanisms[49]. During the seedling stage, LTS can delay rice development and even cause seedling mortality[50], while during the vegetative growth stage, it reduces yield by decreasing biomass accumulation[47]. During the jointing stage, LTS decreases rice yield by reducing the number of spikelets per panicle[51]. During the booting stage, the exposure to LTS can lead to the occurrence of male sterility, a phenomenon that subsequently leads to a significant decrease in the number of spikelets within each panicle. The reproductive characteristics of rice are ultimately influenced by the direct effect of LTS, thereby affecting the final yield and quality[52, 53]. During the heading and flowering stage, LTS can hinder the natural dehiscence of anthers, subsequently compromising the process of pollination. As a result, this leads to a significant decrease in pollen fertilization rates and a substantial increase in the proportion of empty hulls[51, 54].

However, The impact of low-temperature stress (LTS) on rice cultivated under natural field conditions was significantly distinct from that observed in rice grown under laboratory-controlled conditions [55]. Previous studies on rice LTS have predominantly focused on constant low-temperature conditions or limited temperature gradient treatments during specific growth stages. However, hourly dynamic low-temperature simulation based on natural diurnal rhythms has not been adequately addressed in the existing literature. Therefore, this study designed and implemented a rice LTS experiment replicating the low-temperature conditions experienced under natural field conditions. Based on a two-year LTS experiment, with rice growth and development under natural conditions serving as the control, this study comparatively analyzed the effects of hourly dynamic LTS on growth, physiological and biochemical characteristics, and yield at different growth stages of rice. These findings could provide a theoretical basis for identifying and evaluating LTS in rice under field conditions, thereby holding significant practical implications.

2. Materials and Methods

2.1. *Experimental Design*

Controll[15]ed-environment LTS phytotron experiments were conducted in 2023 and 2024 at Rice Research Institute of Heilongjiang Academy of Agricultural Sciences in Jiamusi (46.87°N and 130.53°E), Heilongjiang Province, China. A Japonica inbred rice cultivar, Longgeng31, with moderate cold tolerance was selected for the experiment. Seedlings at three-leaf stage were transplanted into plastic pots (diameter 28 cm and height 45 cm) filled with 15 kg dry paddy

soil that sourced from the experimental farm of Rice Research Institute at Heilongjiang Academy of Agricultural Sciences. The soil exhibited the following basic physical and chemical properties: pH of 6.22, organic matter content of 36.56 g/kg, alkali hydrolysis nitrogen content of 105.35 mg/kg, available phosphorus content of 82.56 mg/kg, and available potassium content of 92.6 mg/kg. After undergoing disinfection, seed soaking, and germination processes, the seeds of Longgeng31 were sown on a seedling plate containing paddy soil and cultivated in a plastic shed. When the rice seedlings reached the stage of having three leaves and one heart leaf, seedlings with similar developmental stages were carefully selected and transplanted into plastic pots. The planting density was 3 hills per pot (3 seedlings per hill) for a total of 90 pots. The planting density was 3 hills per pot (3 seedlings per hill). All the pots were grown under ambient weather conditions before LTS treatment. 2 g ammonia sulphate, 1 g potassium sulphate, and 1 g diammonium phosphate were applied in each pot as the basal fertilizer, and an additional 1 g ammonia sulphate was applied at the panicle differentiation stage of rice. The pots were kept flooded until one week before harvesting. Watering at the late active tillering stage was stopped to ensure efficient tillering. Weeds were removed manually, whereas pest and diseases were controlled using pesticides.

Thirty-six pots of each low-temperature treatment were transferred into the two phytotrons respectively, once the rice plants reached the targeted tillering, booting, and heading stages. LTS was designed at two temperature levels of T2 and T3 (with daily average temperature at 13.5 °C and 11.5 °C, respectively) at tillering stage, T1 and T2 (with daily average temperature at 17.5 °C and 13.5 °C, respectively) at booting and heading stages (Figure 1), and three temperature durations of D3, D7, D10 (3days, 7days, and 10 days, respectively), with 12 h-light/12 h-dark photoperiod, 70% RH.

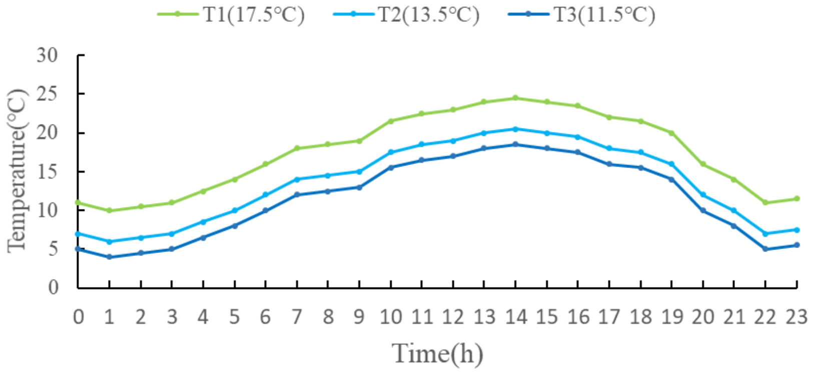

Figure 1. The day-night temperatures in the phytotron.

In this study, rice subjected to low-temperature treatment was compared with rice grown under natural conditions, which served as the control group (CK). This comparison aimed to analyze the effects of varying intensities of LTS on the growth and development of different rice growth stages. The average daily mean temperature for rice cultivated under natural conditions in 2023 and 2024 was utilized as the reference average temperature for the CK in this study, and the temperature conditions for each CK are presented in Table 1.

Table 1. The average daily mean temperature, average daily minimum temperature, and average daily maximum temperature of each CK group of cultivated rice grown under natural conditions in 2023 and 2024.

Growth stage	Duration of LTS treatment	Average daily mean temperature(°C)	Average daily minimum	Average daily maximum

		(CK)	temperature(°C)	temperature(°C)
Tillering stage	D3	18.2	15.1	21.2
	D7	20.4	15.8	24.8
	D10	21.2	16.9	25.7
Booting stage	D3	19.9	16.0	24.2
	D7	23.3	18.4	28.2
	D10	23.6	19.0	28.1
Heading stage	D3	24.3	19.5	29.0
	D7	24.7	20.3	29.4
	D10	23.9	20.4	28.1

Upon the completion of each low-temperature treatment for the designated duration, twelve pots of the rice were transferred outdoors to resume growth under natural conditions until they achieved full maturity.

2.2. LTS treatment and sample preparation

At the tillering stage, when the tillering ratio of rice plants reached 50%, 12 pots exhibiting uniform growth were carefully selected and transferred to individual phytotrons for LTS treatment. The LTS treatment applied during the booting and heading stages was identical to that administered during the tillering stage. After subjecting the pots to LTS treatment for 3, 7, and 10 days (12 h-light/12 h-dark photoperiod, 80% RH) at each growth stage, they were subsequently transferred to an outdoor natural environment for cultivation until reaching maturity. The flag leaf samples were collected from each group at T1, T2, and T3 after 3 days, 7 days, and 10 days of treatment respectively with a total weight of 0.5 g for each sample. The fresh flag leaf samples were promptly flash-frozen in liquid nitrogen and subsequently stored at a temperature of -80 °C.

2.3. Yield and its components

After the plants reached maturity, nine holes rice plants (3 pots) were randomly selected from each treatment. The plants were harvested from the base on the soil surface. The sample plants were first separated into green leaves, dead leaves, stems and panicles and then dried at 80 °C for 72 hr or until the weight stopped decreasing. The harvested plants from each replication were used to determine grain yield and yield components. Grain Yield (g per plot) and yield components as 1000-grain weight (g), Seed set percentage (%), grain number per panicle, and effective panicles (number per pot) were calculated after harvesting at physiological maturity as previously described[56].

2.4. Photosynthetic parameters

The photosynthetic characteristics of the flag leaf were measured within 2-3 days after each LTS treatment, with a total of 9 repetitions per treatment. The changes of net photosynthetic rate (P_n), intercellular CO_2 concentration (C_i), stomatal conductance (G_s) and transpiration rate (T_r) on the fully expanded flag leaves of rice plants under different LTS treatments were measured using a portable type Li-6400 (Li-6400, Li-Cor Inc., USA) plant photosynthetic measurement apparatus between 9:30–11:30. The analyses were performed under an air flow rate of 200μmol·s–1, 65% humidity, 350μmol (photon)·m^{-2}·s^{-1} light intensity, and about 350 μmol·mol–1 ambient CO_2 concentration[57]. The analyses were conducted under an air flow rate of 200 μmol·s^{-1}, a humidity level of 65%, a light intensity of 350 μmol (photon)·m^{-2}·s^{-1}, and an ambient CO_2 concentration of approximately 400 μmol·mol^{-1}.

2.5. Determination of the content of ROS and lipid peroxides

The MDA content was determined by the reaction with thiobarbituric acid (TBA)[58]. The absorbance of MDA was measured at 440, 532, and 600 nm using UV–Vis Spectrophotometer (Mettler-Toledo UV5Bio, Switzerland).

The superoxide (O_2^-) content was determined following the method of Batool et al[59]. The phosphate buffer (pH 7.8, 0.5 mL) was mixed with p-aminobenzene sulfonic acid (17 mM, 1 mL), hydroxylammonium chloride (1 mM, 1 mL), and α-naphthylamine (7 mM, 1.0 mL). The reaction mixture was incubated at a temperature of 25 °C for a duration of 60 minutes and subsequently measured for absorbance at a wavelength of 530 nm.

The H_2O_2 content was determined as described by Song et al[60]. The samples were homogenized with trichloroacetic acid (0.1%) and subsequently centrifuged at 10,000×g at 4 °C for 20 minutes. Afterwards, 1 mL of the resulting supernatant was combined with 2 mL of KI (1 M) and 1 mL of K_2PO_4 buffer, followed by measuring the absorbance at a wavelength of 390 nm after subjecting to darkness treatment for one hour.

EL was calculated by referring to the experimental analysis method of Li et al [61]. Each fresh 0.5 g leaf of rice was placed in individual stoppered triangular flasks containing 10 mL of deionized water. The samples were incubated at 25 °C for 24 h. The electrical conductivity of the solution (S1) was measured after incubation. The samples were then placed in a boiling water bath for 10 min and the second measurement (S2) was determined after cooling the solutions to room temperature. EL = S1/S2 × 100%.

2.6. Determination of antioxidant enzyme activity.

The frozen flag leaf samples were homogenized with a pre-cooled pestle and mortar in potassium phosphate buffer (50 mM, pH 7.8) supplemented with 1% polyvinylpyrrolidone. The prepared crude enzyme extract was incubated with the supernatant obtained after homogenization and subsequently centrifuged at 15,000×g at 4 °C for 20 min. The activities of POD, SOD, and CAT were measured following the method of Xu et al[62].

2.7. Determination of the contents of osmotic adjustment substances.

The estimation of free proline was conducted according to the method proposed by Bates et al. with minor modifications[63]. The samples were immersed in 5 mL of sulphosalicylic acid (3%, 100 °C for 10 minutes). Subsequently, a volume of 2 mL from the resulting extract was combined with ninhydrin reagent containing glacial acetic acid and incubated at 100 °C for a duration of 30 minutes. After being cooled in ice water, 4 mL of toluene was added to the mixture and subsequently measured at a wavelength of 520 nm for the determination of proline content. The quantification of SP content was performed using the BCA method according to the established protocol by Campion et al[64]. The quantification of soluble sugar content was determined following the experimental analysis protocol established by Loutfy et al[65].

2.8. Preparation of chloroplast electron microscope samples

The first wholly unfolded leaf at the top of the rice of different treatments was cut into 2 mm^2 miniature pieces with a sharp double-sided knife (at the same position on the leaf) and placed in a refrigerator at 4 °C with 2.5% glutaraldehyde for pre-fixation. The electron microscope samples, fixed with glutaraldehyde, were subjected to three rounds of cleaning with PBS (0.1 mol/L, pH 6.8) at 15-minute intervals. After fixation with osmium tetroxide for 1.4 hours, the samples were subjected to three rounds of PBS cleaning for 10 minutes each. Following the cleaning process, the samples underwent dehydration using a gradient of ethanol concentrations (50%, 70%, 90%, and 100%), with each concentration being applied twice for a duration of 10 minutes per application. The dehydrated samples were immersed in a 1:1 mixture of 100% ethanol and 100% acetone for a duration of 10 minutes, followed by

immersion in pure acetone for 5 minutes. Subsequently, the samples were soaked in a solution containing 100% acetone and Epon812 resin (in a ratio of 1.0:1.5) for an hour. After complete embedding in pure resin at a temperature of 30 °C for 5 hours, the polymerization process was initiated with a gradual increase in temperature to reach between 36-48 hours at 60 °C. Finally, the samples were embedded using pure embedding agent until the following day and sliced using an LKB5 micro-slicing machine before being observed and photographed under Hitachi H7650 transmission electron microscopy.

2.9. Determination of the Pollen fertility.

On the day of rice flowering, three representative panicle spikelets were selected from each treatment and three anthers were collected from each spikelet for analysis. The flowers were stained with I_2-KI solution and placed under a stereomicroscope to observe the staining of three visual fields and determine the fertility of pollen grains.

2.10. Statistical analyses.

In this study, the average of two years' test results for each treatment was selected for analysis to ensure the repeatability and reliability of the low-temperature stress test outcomes. All the data obtained were statistically analyzed using the SPSS 27.0 (SPSS Inc., Chicago, IL, USA) for variance (ANOVA) analysis. The least significant difference (LSD) test was used to determine the significant differences among treatments ($P < 0.05$).

3. Results

3.1. Effect of LTS on Photosynthetic enzymes

The LTS treatment at the tillering, booting, and heading stages reduced P_n of rice leaves (Figure 2). P_n showed a decrease as the LTS temperature decreased and the LTS duration increased. At T3, P_n was most reduced by LTS treatment at booting stage, followed by the tillering stage, and the heading stage. At tillering stage, with the increase in LTS duration, the LTS treatment at T2 and T3 significantly reduced P_n by 52.2% ~ 62.7%, and 65.9% ~ 78.3% compared with CK, respectively (Figure 2a). At booting stage, there was no significant decrease in P_n when the LTS duration was increased at T1 from D3 to D7, but P_n showed a significant decrease by 29.6% of D10. And, there was significant decrease in P_n when the LTS duration was increased at T3 from D7 to D10, by 85.3%, and 93.9% compared with CK, respectively (Figure 2b). At heading stage, with the LTS duration increased, P_n showed a non-significant decrease at T1, but a significant decrease at T3 by 39.3% ~ 44.9% compared with CK (Figure 2c).

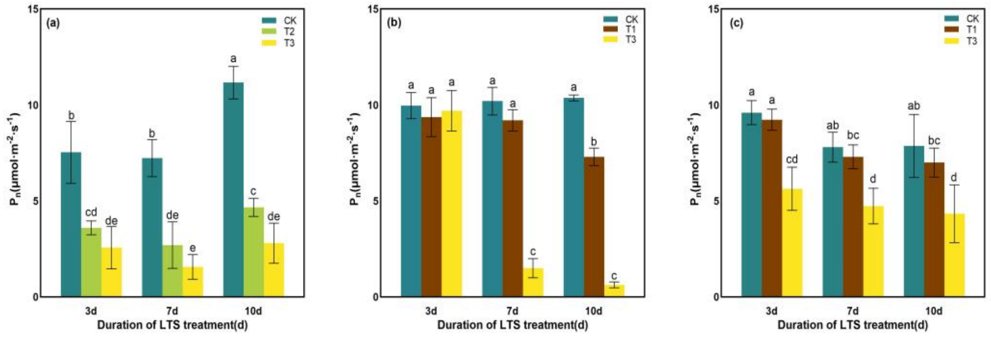

Figure 2. Effects of LTS on P_n. (a) The variation trend of the tillering stage. (b) The variation trend of the booting stage. (c) The variation trend of the heading stage. Notes: The values represent the mean ± SE (n

= 4). Mean values provided with error bars representing the standard error. Different letters denote statistical differences at $p < 0.05$.

C_i increased under the LTS treatment, and its became more pronounced as the LTS temperature decreased (Figure 3). At the tillering stage, C_i initially increased and subsequently decreased as the duration of LTS treatment extended, and the increase of C_i reached the highest at T3 of D3, which was preeminently resulted in the higher accumulation of 25.26% relative to control (Figure 3a). At booting stage, C_i elevated gradually with the decrease of treatment temperature while the duration of low-temperature treatment was the same. C_i of T3 treatment was 4.97% ~ 39.88% higher than that of T1 treatment (Figure 3b). There was no effective change in C_i of 3d leaves treated by T1 at heading stage, while there was noted difference in other treatments compared to those in the control treatments at the same time. Compared with CK, C_i was memorably elevated by 13.82% and 19.91% at each temperature for the duration of D10 (Figure 3c).

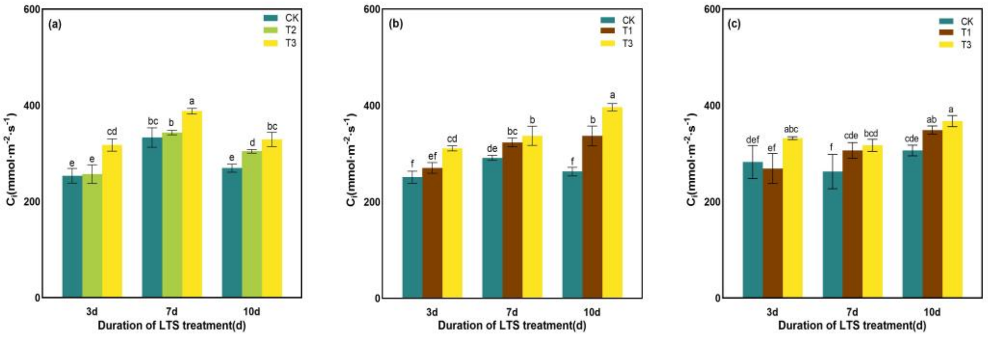

Figure 3. Effects of LTS on Ci. (a) The variation trend of the tillering stage. (b) The variation trend of the booting stage. (c) The variation trend of the heading stage. Notes: The values represent the mean ± SE (n = 4). Mean values provided with error bars representing the standard error. Different letters denote statistical differences at $p < 0.05$.

Under LTS, with the decrease of temperature, G_s showed a decreasing trend under all LTS treatments, and the decrease was most obvious at T3. At T3, with the increase of LTS days, G_s decreased by 65.88%~86.10%, 22.59%~81.00% and 20.94%~67.71%, respectively, compared with CK in the same period (Figure 4). At the tillering stage, G_s initially decreased and then increased with the duration of LTS treatment days, and most significant decrease of G_s occurred on the 7th day compared to the CK (Figure 4a). At the booting stage from 7 to 10 days, with the decrease of low-temperature, G_s decreased gradually and the difference between treatments was significant (Figure 4b). The same performance was also observed when LTS treatment was carried out for 3 days at heading stage (Figure 4c).

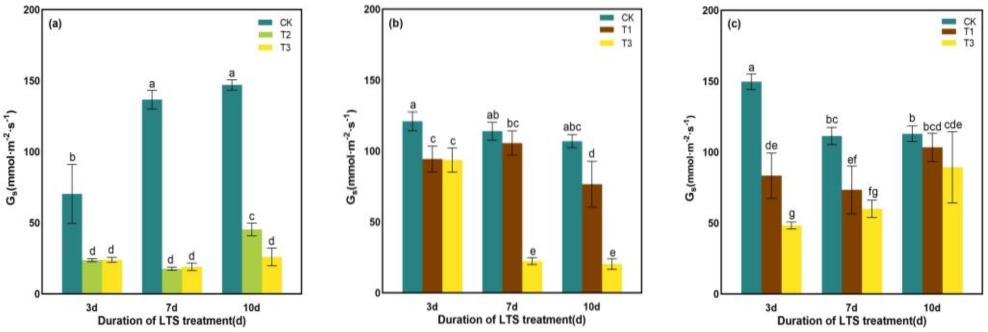

Figure 4. Effects of LTS on G_s. (a) The variation trend of the tillering stage. (b) The variation trend of the booting stage. (c) The variation trend of the heading stage. Notes: The values represent the mean ± SE (n = 4). Mean values provided with error bars representing the standard error. Different letters denote statistical differences at $p < 0.05$.

At tillering stage, T_r decreased by low-temperature, and it decreased by 42.04%~68.75% and 37.70%~78.13%, respectively, compared with CK treatment (Figure 5a). At booting stage, the T_r of leaves gradually decreased with the decrease of treatment temperature under the same treatment days. In comparison to control plants, T_r was spanking reduced by 47.32% and 84.94% after 10d of LTS treatment (Figure 5b). At heading stage, the T_r of rice leaves increased first and then decreased with the increase of LTS treatment duration. The LTS treatment at T1 strikingly reduced T_r by 33.33% compared with the LTS treatment at T3, for the LTS treatment duration of D3 (Figure 5c).

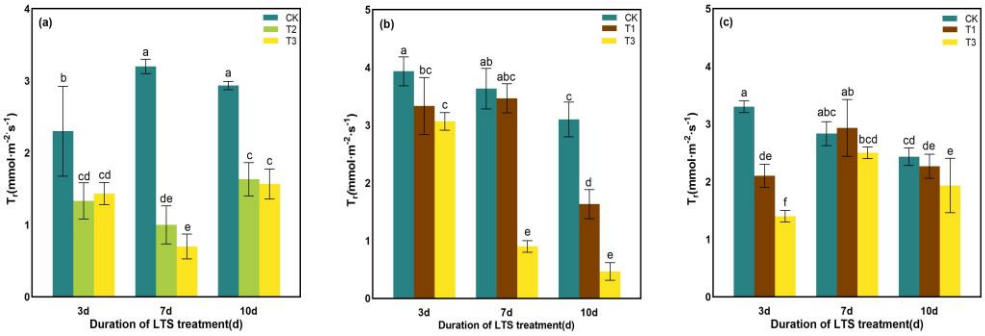

Figure 5. Effects of LTS on T_r. (a) The variation trend of the tillering stage. (b) The variation trend of the booting stage. (c) The variation trend of the heading stage. Notes: The values represent the mean ± SE (n = 4). Mean values provided with error bars representing the standard error. Different letters denote statistical differences at $p < 0.05$.

3.2. Effect of LTS on Oxidative Stress Indicators

MDA content of leaves changed significantly after low-temperature treatments. The content of MDA increased with the decrease of treatment temperature and the extension of treatment time, and T3 treatments prompted to maximum MDA content at 10 days. Compared with CK, MDA content at different periods was notedly elevated by 119.87%, 88.37% and 84.09% under T3 treatments, respectively (Figure 6).

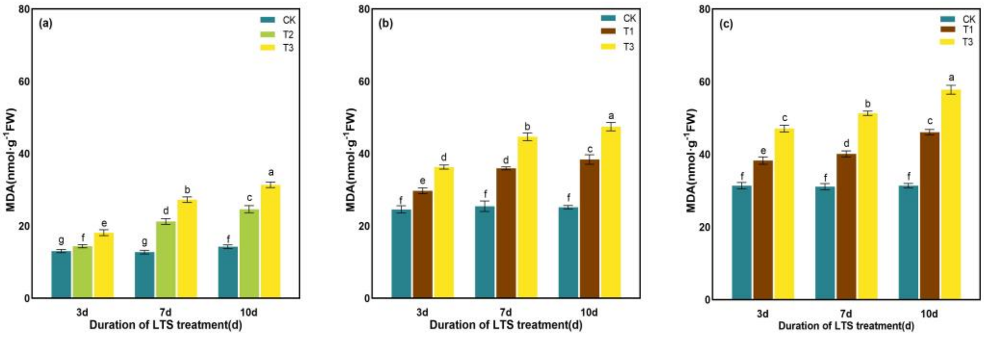

Figure 6. Effects of LTS on MDA. (a) The variation trend of the tillering stage. (b) The variation trend of the booting stage. (c) The variation trend of the heading stage. Notes: The values represent the mean ± SE (n = 4). Mean values provided with error bars representing the standard error. Different letters denote statistical differences at $p < 0.05$.

EL of rice leaves was much exacerbated when plants were exposed to low-temperature. Compared with normal temperature treatment, EL particularly increased by 36.51%, 61.81% and 95.04%, respectively, under T2 treatment on 3~10 days at tillering stage (Fig. 6a). After 10 days of treatment, the maximum value was 2.14 times, 2.41 times and 2.36 times of the control treatment under T3 treatments, respectively (Figure 7).

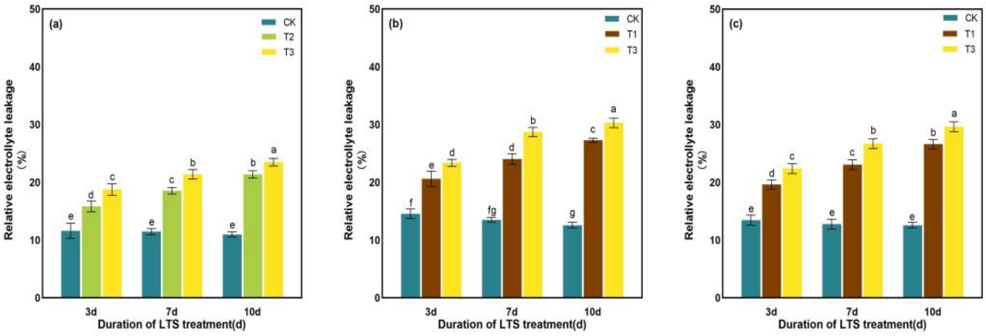

Figure 7. Effects of LTS on EL. (a) The variation trend of the tillering stage. (b) The variation trend of the booting stage. (c) The variation trend of the heading stage. Notes: The values represent the mean ± SE (n = 4). Mean values provided with error bars representing the standard error. Different letters denote statistical differences at $p < 0.05$.

Low-temperature stress promoted the accumulation of O_2^- in rice, and the production rate of O_2^-in the plants treated with low-temperature was always higher than that in the control group. The production rate of O_2^-increased gradually with the extension of stress time. Compared with the control group, the production rate of O_2^- was significantly increased by 91.21%, 66.88% and 42.48% after 10 days of T3 low-temperature treatment, respectively. It was significantly higher than 10.19%, 7.30% and 6.32% on 7d (Figure 8).

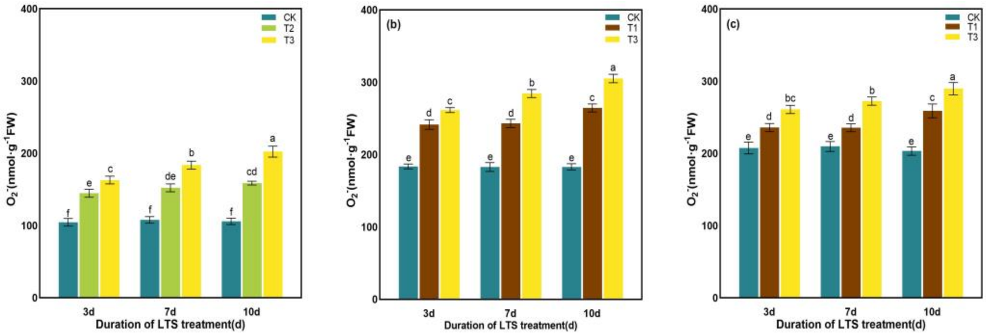

Figure 8. Effects of LTS on the production rate of O_2^-. (a) The variation trend of the tillering stage. (b) The variation trend of the booting stage. (c) The variation trend of the heading stage. Notes: The values represent the mean ± SE (n = 4). Mean values provided with error bars representing the standard error. Different letters denote statistical differences at $p < 0.05$.

The change of H_2O_2 content in different periods under low-temperature stress was measured. The results showed that the H_2O_2 content of rice under low-temperature treatment aggrandized significantly from 3 to 10 days, and was significantly higher than that of the control. With the advance of stress time, the change of H_2O_2 content showed an increasing trend, and reached the peak value at 10 days after T3 treatment, which increased by 244.55% compared with that under no stress. 122.09% and 137.03%. The H_2O_2 content increased the most after 10 days of treatment (Figure 9).

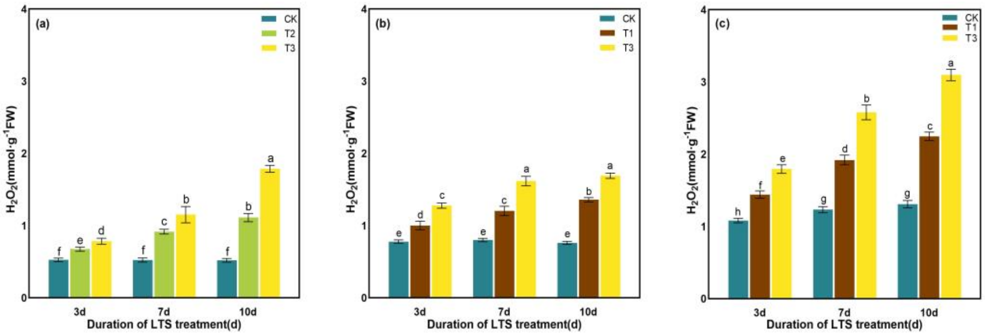

Figure 9. Effects of LTS on H_2O_2. (a) The variation trend of the tillering stage. (b) The variation trend of the booting stage. (c) The variation trend of the heading stage. Notes: The values represent the mean ± SE (n = 4). Mean values provided with error bars representing the standard error. Different letters denote statistical differences at $p < 0.05$.

3.3. Effect of LTS on Antioxidant Enzyme Activity

As can be seen from Figure 10, rice leaves maintained a higher accumulation of SOD activity at the tillering stage at T2 and T3, and augmented conspicuously compared with that at room temperature. Under the same treatment days at booting stage and heading stage, SOD activity of rice leaves could be supplemented with the decrease of temperature, compared with normal level. In different time stages, SOD activity in rice leaves increased first and then decreased with the extension of time, and reached the maximum value on the 7th day under T3 treatment, which a particularly improvement by 17.11%, 15.12% and 19.31% relative to those in the non-stressed plants, respectively.

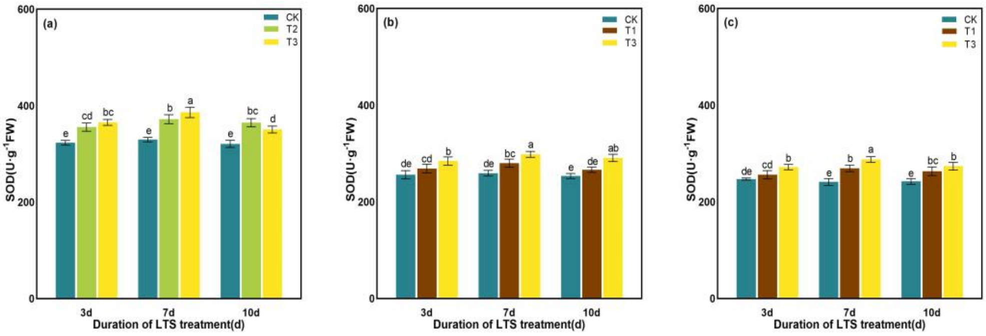

Figure 10. Effects of LTS on SOD. (a) The variation trend of the tillering stage. (b) The variation trend of the booting stage. (c) The variation trend of the heading stage. Notes: The values represent the mean ± SE (n = 4). Mean values provided with error bars representing the standard error. Different letters denote statistical differences at $p < 0.05$.

The POD activity of rice leaves showed the trend of "unimodal curve", and reached the peak value on the 7th day after the two low-temperature treatments (Figure 11). On tillering stage, POD activity of T2 treatment was evidently elevated by 5.81%, 9.91% and 7.67% relative to control at 3d~10d (Figure 11a). At booting stage and heading stage, POD activity augmented with the increase of treatment days, and T3 treatment preeminently accumulated by 1.87% to 7.01% compared with T1 (Figure 11b).

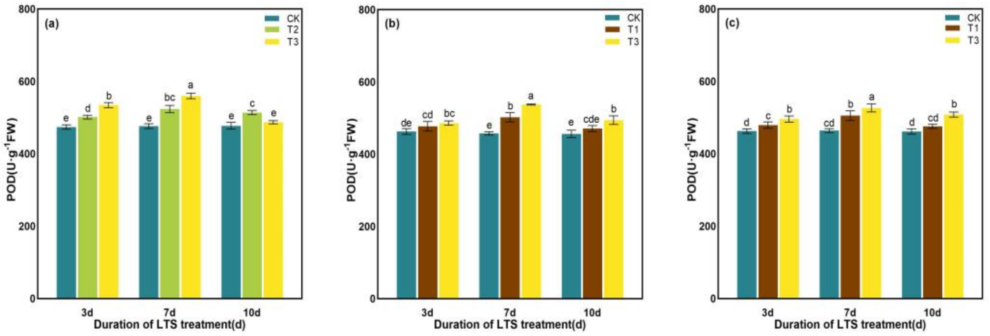

Figure 11. Effects of LTS on POD. (a) The variation trend of the tillering stage. (b) The variation trend of the booting stage. (c) The variation trend of the heading stage. Notes: The values represent the mean ± SE (n = 4). Mean values provided with error bars representing the standard error. Different letters denote statistical differences at $p < 0.05$.

At tillering stage, the CAT enzyme activity of rice leaves showed a notable decline trend after 10 days of T2 and T3 treatment, which decreased by 11.36% and 27.32%, respectively, compared to those in the control treatments (Figure 12a). Under the same low-temperature treatment, the increase of activity after 7 days of low-temperature treatment was higher than that after 3 days and 10 days of low-temperature treatment, which spanking enhanced by 13.11% and 21.74%, 16.12% and 28.81%, 14.97% and 23.77, respectively, compared with the same treatment days in the same period (Figure 12).

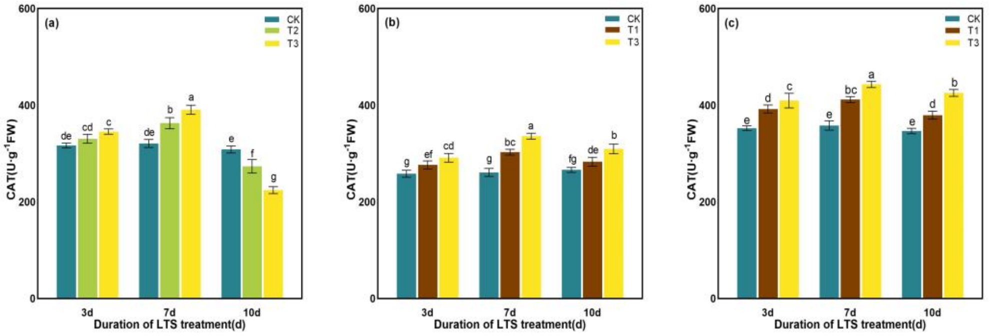

Figure 12. Effects of LTS on CAT. (a) The variation trend of the tillering stage. (b) The variation trend of the booting stage. (c) The variation trend of the heading stage. Notes: The values represent the mean ± SE (n = 4). Mean values provided with error bars representing the standard error. Different letters denote statistical differences at $p < 0.05$.

3.4. Effect of LTS on Osmolyte Contents

LTS resulted in the higher accumulation of proline in rice at different stages. However, the proline content in rice leaves showed no significant alteration under low-temperature stress during the tillering stage at T2 on D3, the booting stage at T1 on D10, and the heading stage at T1 on D3. Under the same temperature treatment, the proline content increased first and then decreased with the extension of days, reaching a peak on D7. In the same days of treatment, the proline content increased gradually with the decrease of temperature, except for 10 days of T3 treatment at tillering stage (Figure 13). For example, during the booting stage of rice, proline content continued to remarkably increase with decreasing temperature, reaching a peak at T3. The content of proline preeminently accumulated by 13.10% and 48.66% in comparison with CK treatment on D7.

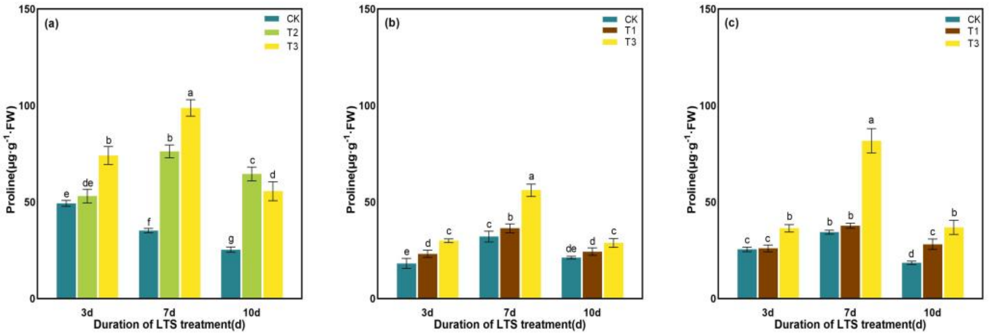

Figure 13. Effects of LTS on proline. (a) The variation trend of the tillering stage. (b) The variation trend of the booting stage. (c) The variation trend of the heading stage. Notes: The values represent the mean ± SE (n = 4). Mean values provided with error bars representing the standard error. Different letters denote statistical differences at $p < 0.05$.

With the extension of low-temperature treatment time, the soluble sugar content of rice showed a unimodal curve in different growth stages. The maximum value was reached after 7 days of low-temperature treatment. The change of soluble sugar content was different with different days of low-temperature stress. The content of soluble sugar was spanking higher after 7 days of low-temperature treatment at the booting stage than other treatments, which was particularly increased by 59.25% and 100.52% compared with the control, respectively (Figure 14).

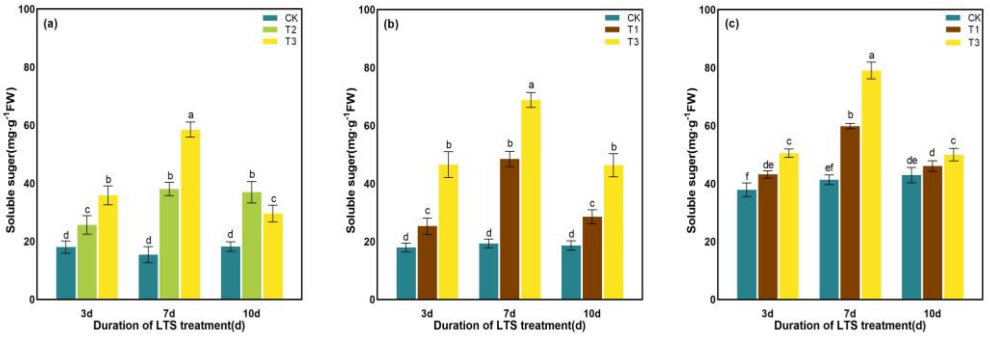

Figure 14. Effects of LTS on the soluble sugar. (a) The variation trend of the tillering stage. (b) The variation trend of the booting stage. (c) The variation trend of the heading stage. Notes: The values represent the mean ± SE (n = 4). Mean values provided with error bars representing the standard error. Different letters denote statistical differences at $p < 0.05$.

After low-temperature treatment, the content of soluble protein in rice leaves increased gradually (Figure 15). At the tillering stage, the low-temperature treatment at 3d, 7d and 10 d increased by 1.56%, 2.66% and 2.65% compared with CK treatment at T2 (Figure 15a). At the heading stage, low-temperature treatment for 3d, 7d and 10d, T3 treatment was significantly increased by 6.03%, 9.42% and 6.68% compared with CK, respectively (Figure 15c). The contents of soluble protein in rice leaves reached the maximum value under T3 stress at 7 days, increasing by 5.54%, 12.06% and 9.42% compared with CK at the same growth stage, respectively ($P<0.001$).

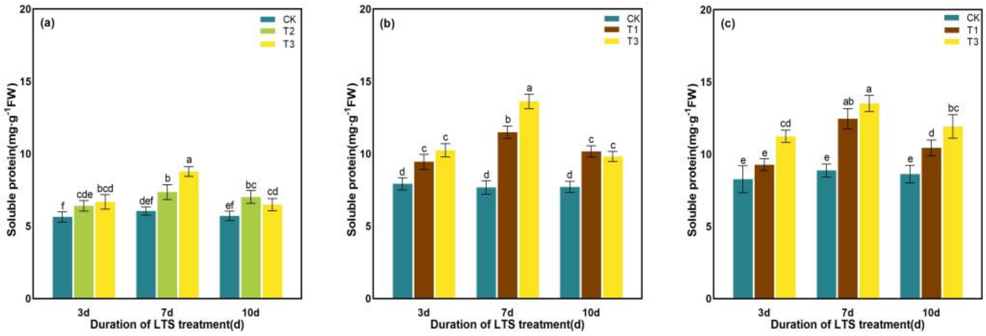

Figure 15. Effects of LTS on the soluble protein. (a) The variation trend of the tillering stage. (b) The variation trend of the booting stage. (c) The variation trend of the heading stage. Notes: The values represent the mean ± SE (n = 4). Mean values provided with error bars representing the standard error. Different letters denote statistical differences at $p < 0.05$.

3.5. Effect of LTS on Pollen Viability

With the prolongation of low-temperature treatment duration, pollen viability decreased gradually in each period (Figure 16). At the tillering stage, there were significant differences between CK and T3 treatment at day 7 and 10, which considerably reduced by 6.30% and 6.63% compared with normal temperature conditions at the same stage (Figure 16a). At booting stage, pollen viability of 10-day low-temperature treatment decreased the most, a sharp decrease of about 30.04% and 31.46% was observed compared to control (Figure 16b). Under T1 treatment, pollen viability suppressed particularly with the extension of days. At heading stage, under the same low-temperature treatment, there was dramatically difference between 10-day and 3-day treatment, but no evident difference between 7-day treatment (Figure 16c).

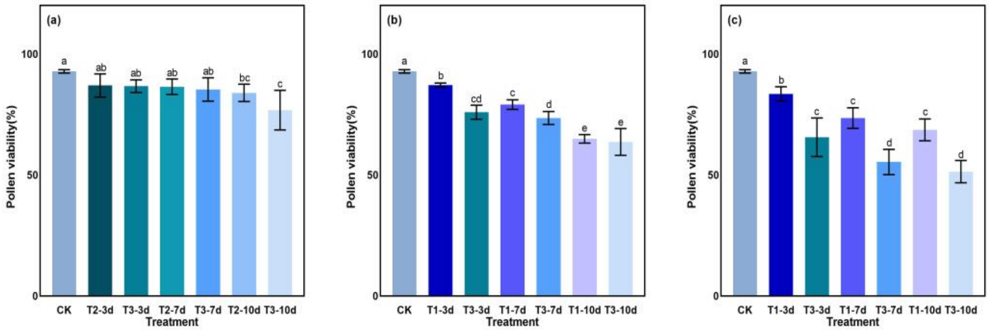

Figure 16. Effects of LTS on pollen viability. (a) The variation trend of the tillering stage. (b) The variation trend of the booting stage. (c) The variation trend of the heading stage. Notes: The values represent the mean ± SE (n = 4). Mean values provided with error bars representing the standard error. Different letters denote statistical differences at $p < 0.05$.

3.6. Yield and its components

Grain yield and related components were significant reduced under LTS at the tillering, booting, and heading stages. At the tillering stage, seed set percentages exhibited reductions of 2.25% and 9.80%, 7.47% and 15.21%, as well as 11.04% and 21.12% for durations of 3, 7, and 10 days at T2 and T3 in comparison to CK, with significant differences noted except for the T2 treatment at the three-day interval. Additionally, grain yield significantly decreased by 21.62% and 36.59%, 25.57% and 47.80%, as well as by 37.89% and 51.89%, corresponding to the same time frames at T2 and T3 when compared to CK. At booting stage, compared with CK, the percentage reductions observed were 29.65%and 37.70%, 30.14% and 39.34%, as well as 41.19%

and 46.89% for grains per panicle, 15.48% and 60.09%, 18.72% and 77.13%, as well as 59.30% and 88.76% for grain yield, under low-temperature stress conditions T1 and T3, with durations of 3, 7, and 10 days respectively, all showing significant variations. At heading stage, under low-temperature stress of T1, grains per panicle, and grain yield were significantly decreased by 21.52%, 29.08% and 31.50%, as well as 14.69%, 36.42% and 53.01, for durations of 3, 7, and 10 days in comparison to the control, respectively. Furthermore, compared with CK, grains per panicle, and grain yield were significantly decreased by 25.13%, 34.73% and 39.51%, as well as 42.03%, 51.45% and 74.84%, for durations of 3, 7, and 10days, under low-temperature stress conditions T3, respectively (Table 2).

Table 2. Effect of low-temperature stress on grain yield and its components at different growth stages.

Growth stages	Treatment	Treatment days/d	Effective panicles (number/pot)	Grain/panicle	Seed set percentage (%)	1000-grain weight (g)	Grain yield (g/pot)
Tillering stage	CK		17.67±2.52a	170.67±14.5a	89.97±1.46a	22.81±0.27a	37.47±6.6a
	T2	3	15.67±2.08a	155±12.77ab	87.95±1.61a	22.66±0.28a	29.37±0.85b
	T3		13.67±4.73a	153.67±7.02ab	81.16±1.77b	22.61±0.32a	23.76±1.22bcd
	T2	7	14±2a	151.33±9.02ab	83.25±2.4b	22.47±0.17ab	27.89±0.98bc
	T3		12±3.61a	149.33±13.05ab	76.29±2.31c	22.25±0.57ab	19.56±2.37d
	T2	10	13.67±0.58a	145.67±11.5ab	80.04±2.92b	22.7±0.52a	23.27±0.94cd
	T3		11.33±4.04a	144±15.52b	70.97±2.03d	21.78±0.37c	18.03±3.96d
Booting stage	CK		17.67±2.52a	170.67±14.5a	89.97±1.46ab	22.81±0.27a	37.47±6.6a
	T1	3	16.33±1.53a	120.07±5.42b	93.58±1.13a	22.42±0.34ab	31.67±2.74a
	T3		14.33±2.52ab	106.33±3.05bc	64.29±2.99d	21.76±0.58b	14.95±3.85b
	T1	7	16±3.61a	119.23±5.03b	85.78±2.42b	22.28±0.39ab	30.45±5.5a
	T3		10.33±1.53bc	103.53±1.79cd	40.48±3.41e	20.76±0.9cd	8.57±0.77bc
	T1	10	13±3.61ab	100.37±7.46cd	70.94±4.23c	21.51±0.48bc	15.25±3.5b
	T3		7.67±1.53c	90.63±10.02d	21.9±2.48f	20.51±0.6d	4.21±1.91c
Heading stage	CK		17.67±2.52a	170.67±14.5a	89.97±1.46a	22.81±0.27a	37.47±6.6a
	T1	3	17.33±1.53ab	133.93±5.69b	94.17±1.17a	22.77±0.88a	31.96±6.38a
	T3		13±2.65c	127.77±5.49bc	67.98±3.63bc	22.31±1.16ab	21.72±5.12b
	T1	7	13.67±2.52bc	121.03±7.17bcd	73.68±2.12b	22.6±0.55a	23.83±1.25b
	T3		12.33±2.52c	111.4±8.41de	63.19±6.88cd	21.52±0.93ab	18.19±2.28b
	T1	10	12±1c	116.9±6.31cde	57.81±3.38d	21.6±0.58ab	17.61±2.1b
	T3		11.33±1.53c	103.23±9.23e	44.88±8.37e	20.81±1.08c	9.43±1.71c

CK, under the natural conditions (control); T1, low-temperature stress of mean temperature 17.5°C; T2, low-temperature stress of mean temperature 13.5°C; T3, low-temperature stress of mean temperature 11.5°C.

4. Discussion

4.1. Physiological and biochemical properties of cold-region japonica rice

Low-temperature is one of the most important adversities affecting crop growth, development and yield[22]. Cold-region japonica rice plays an important role in rice production in China, and its special geographical location and climate change lead to frequent low-temperature and cold injury[14]. This study found that continuous low-temperature would reduce plant height, and stress response to low-temperature in different periods was the same, and in the same period of the same days, the lower the temperature, the more obvious the decline of rice plant height. Rice plant height decreased in the early stage of low-temperature in different periods, but the difference was not significant. With the extension of

time, the decrease of rice plant height was greater. The results showed that the inhibition effect of low-temperature for a long time on rice growth was more obvious under certain temperature conditions.

Under normal temperature, plants can automatically regulate the production and removal of reactive oxygen species (ROS), so that they are in a state of dynamic equilibrium in the body. However, under low-temperature conditions, plant growth is inhibited, resulting in excessive accumulation of ROS in leaves and aggravation of membrane lipid peroxidation[62]. Plants use SOD, POD and other antioxidant enzymes to maintain the balance of active oxygen metabolism, effectively remove excess active oxygen in cells, and reduce the damage to plant cell membranes[66]. SOD, as an important active enzyme in antioxidant process, has the property of scavenging superoxide free radicals. POD is a kind of oxidoreductase produced by plants, which can eliminate the toxicity of ROS, H_2O_2, phenols and amines produced by plants, and reduce the damage of reactive oxygen species to plants. CAT can remove H_2O_2 produced by stress in plants and decompose the accumulated H_2O_2 into H_2O and O_2, thereby reducing its oxidative damage to plant tissues[67]. When plants are subjected to low-temperature stress, they will also suffer from osmotic stress and water stress. The contents of some osmotic regulatory substances with water-retaining effect, such as soluble sugar, soluble protein and proline, will increase to maintain the osmotic balance of cells and reduce the damage to crops caused by low-temperature[68]. Soluble protein content is positively correlated with the cold resistance of rice, and the higher the soluble protein content is, the stronger the cold resistance of rice[69]. In addition, soluble proteins can gather together with soluble sugars around important organelles such as chloroplasts to protect them from stress damage[69]. In this study, the activities of SOD and POD in rice leaves were significantly enhanced by LTS, and the contents of soluble protein, soluble sugar and proline were also significantly increased, indicating that rice would open its self-protection mechanism and resist the damage of LTS through its own defense system once adverse conditions were formed. The concentrations of the antioxidant and osmotic regulation systems in rice peaked after 7 days of LTS treatment during the tillering, booting, and heading stages, indicating that the stress response of rice to LTS had reached a critical threshold (Figure 10-15).

Photosynthesis is the basis of plant growth and yield formation. Under low-temperature stress, plant photosynthesis will be inhibited[70]. Photosynthesis is a process that exhibits high sensitivity to LTS during crop development, leading to a significant reduction in the P_n of rice. This study demonstrated that P_n decreased with increasing intensity and duration of LTS. Furthermore, the effects of LTS on P_n in rice plants varied across different growth stages, with the booting stage exhibiting the most significant reduction, followed by the tillering stage and then the heading stage, which aligns with previous research findings[23, 71].

The limitation of photosynthesis is generally characterized by stomatal limitation and non-stomatal limitation[72]. Under typical conditions, the opening of stomata results in an increase in stomatal conductance, thereby enhancing intercellular carbon dioxide flux and facilitating a rise in the rate of photosynthesis[70]. Our study found that LTS reduced *Pn*, *Tr*, *Gs* and *Ci* of rice leaves at tillering stage. This occurs because the stomata on rice leaves gradually close, leading to a decrease in stomatal conductance (Gs) and resulting in insufficient CO_2 availability for photosynthesis, which ultimately diminishes the photosynthetic rate. This indicates that stomatal restriction significantly impacts the photosynthesis of rice leaves[73]. Under LTS at booting stage and heading stage, *Pn*, *Tr* and *Gs* of rice leaves decreased, while *Ci* value increased. This may be due to the damage of chloroplast structure in plants under low-temperature stress, which leads to the decreased ability of plant cells to fix and assimilate CO_2, indicating that non-stomatal restriction affects the photosynthesis of rice leaves[22, 74]. Compared with the control group, increasing intensity and prolonged duration of LTS resulted

in distorted chloroplast morphology, further damage to the grana stacking structure within thylakoids, an increase in both size and number of peroxisomes, and a loosening of stroma lamellae. These findings indicate that low-temperature stress inhibits chloroplast biosynthesis, consequently reducing the rate of photosynthesis (Figure 17).

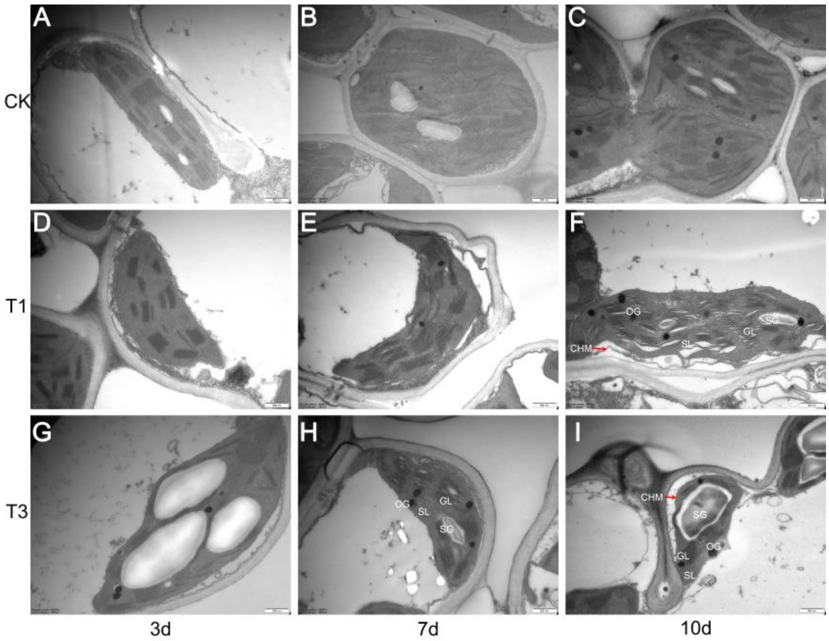

Figure 17. The effect of LTS on chloroplast ultrastructure of rice at booting stage. Chloroplast structure and thylakoid organization in control and LTS were analyzed by transmission electron microscopy (TEM). CK, under the natural conditions (control) for duration of 3, 7 and 10 days (A, B and C); T1, LTS of mean temperature 17.5°C for duration of 3, 7 and 10 days (D, E and F); T3, chilling stress of mean temperature 11.5°C for duration of 3, 7 and 10 days (G, H and I). G: granum; SL: stroma lamellae; GL: grana lamellae; SG: starch grain; OG: osmophilic gramules; CHM: chloroplast membrane. Bar = 500 nm.

4.2. Effects of different growth stages LTS on yields and its components in rice

Yield attribute determines the final yield of rice, the detrimental impact of prolonged low-temperatures on rice yield has been extensively documented in the literature[23, 33, 75, 76]. Previous studies[77, 78] have demonstrated that prolonged low-temperatures during the reproductive phase exert an impact on rice panicle differentiation and flowering, resulting in a decrease in grain weight and seed setting rate, consequently leading to a reduction in rice yield. In this study, LTS reduced rice yield and its components at different growth stages, with the magnitude of decrease exhibiting significant variability. The grain yield, effective panicles, grain number per panicle, 1000-grain weight and seed setting percentage of rice decreased the most at booting stage, followed by heading stage and tillering stage, as the intensity and duration of LTS increased (Table 2). Low-temperature stress during the reproductive phase significantly reduces rice yield, primarily due to a substantial increase in panicle sterility[10, 44]. The results of this study revealed a significant decrease in pollen viability at the booting stage and heading stage under low-temperature stress, when compared to the control group (CK). However, no significant changes were observed in pollen viability at the tillering stage (Figure 16). This offered further substantiation that low-temperature stress at booting stage and heading stage had a greater effect on seed setting rate, with a significantly higher decrease rate compared to that observed at the tillering stage. Moreover, a similar trend was observed in terms of yield.

5. Conclusions

The results showed that plant height and pollen activity reduced substantial under low-temperature stress. MDA, H_2O_2, O_2^- and EL were spanking enhanced. SOD, POD and CAT activities were preeminently augmented. The contents of soluble sugar, soluble protein and proline also elevated dramatically. Low-temperature stress decreases P_n as a result of stomatal limitation during the tillering stage, and non-stomatal limitation during the booting and heading stages. In response to the challenges brought by low-temperature stress, cold resistance can be improved through gene editing or transgenic technology, or by activating specific signal transduction pathways. These measures can effectively improve the cold resistance of rice and other crops under low-temperature stress, reduce the loss caused by low-temperature, and ensure food security.

Author contributions: L.G. and X.D. conceived the original screening and research plans. L.G. and L.J. supervised the experiments. L.G., X.D., J.C., Z.C. and J.L. performed most of the experiments. L.G., J.G., Z.C. and L.J. designed the experiments and analyzed the data. L.G. conceived the project and wrote the article. L.J. supervised and complemented the writing. All authors read and approved the final version of the paper.

Fouding: This research was funded by the National Key Research and Development Program of China (No. 2022YFD2300201), the Natural Science Foundation of Heilongjiang Province (No. LH2024D020) and the Joint Foundation on Regional Meteorological S & T Collaborative Innovation of Northeast China (No. 2024GX006).

Institutional Review Board Statement: Not applicable.

Data Availability Statement: The data presented in this study are available on request from the corresponding author.

Conflicts of Interest: The authors declare no conflict of interest.

References

1. Ito, V.C.;Lacerda, L.G. Black rice (Oryza sativa L.): A review of its historical aspects, chemical composition, nutritional and functional properties, and applications and processing technologies. *Food Chemistry*, 2019, 301.
2. Yuan, S.;Linquist, B.A.;Wilson, L.T.;Cassman, K.G.;Stuart, A.M.;Pede, V.;Miro, B.;Saito, K.;Agustiani, N.;Aristya, V.E.;Krisnadi, L.Y.;Zanon, A.J.;Heinemann, A.B.;Carracelas, G.;Subash, N.;Brahmanand, P.S.;Li, T.;Peng, S.;Grassini, P. Sustainable intensification for a larger global rice bowl. *Nature Communications*, 2021, 12.
3. Hsiaoping, C.;Toriyama, K.;Heong, K.L.;Hardy, B. Rice consumption in China: can China change rice consumption from quantity to quality? in Rice Is Life: Scientific Perspectives for the Century World Rice, Tsukuba,Jepan 2005.
4. Yan, W.;Huang, K. Determinants of agricultural protection in China and the rest of the world. *Asian-Pacific Economic Literature*, 2018, 32, 64-75.
5. Almeida, D.M.;Cecilia Almadanim, M.;Lourenco, T.;Abreu, I.A.;Saibo, N.J.M.;Margarida Oliveira, M. Screening for abiotic stress tolerance in rice: salt, cold, and drought; in Environmental Responses in Plants: Methods and Protocols, P. Duque,eds.; 2016, 1398, 155-182.
6. Teixeira, E.I.;Fischer, G.;van Velthuizen, H.;Walter, C.;Ewert, F. Global hot-spots of heat stress on agricultural crops due to climate change. *Agricultural and Forest Meteorology*, 2013, 170, 206-215.
7. Zheng, E.;Qin, M.;Chen, P.;Xu, T.;Zhang, Z. Climate Change Affects the Utilization of Light and Heat Resources in Paddy Field on the Songnen Plain, China. *Agriculture*, 2022, 12, 1648.
8. Lee, M.H. Low temperature tolerance in rice: the Korean experience. in Increased lowland rice production in the Mekong Region: Proceedings of an International Workshop of the Australian

Centre for International Agricultural Research, Vientiane,Laos 2001.
9. Farrell, T.C.;Fox, K.M.;Williams, R.L.;Fukai, S. Genotypic variation for cold tolerance during reproductive development in rice: Screening with cold air and cold water. *Field Crops Research*, 2006, 98, 178-194.
10. Shimono, H.;Okada, M.;Kanda, E.;Arakawa, I. Low temperature-induced sterility in rice: Evidence for the effects of temperature before panicle initiation. *Field Crops Research*, 2007, 101, 221-231.
11. Shen, X.;Liu, B.;Lu, X. Weak cooling of cold extremes versus continued warming of hot extremes in China during the recent global surface warming hiatus. *Journal of Geophysical Research-Atmospheres*, 2018, 123, 4073-4087.
12. Legg, S. IPCC, 2021: Climate Change 2021: The Physical Science Basis. *Interaction (Melbourne)*, 2021, 49, 44.
13. Tao, F.;Zhang, S.;Zhang, Z. Changes in rice disasters across China in recent decades and the meteorological and agronomic causes. *Regional Environmental Change*, 2013, 13, 743-759.
14. Ji, R.;Yu, W.;Feng, R.;Wu, J.;Zhang, Y. Identification and characteristics of combined agrometeorological disasters caused by low temperature in a rice growing region in Liaoning Province, China. *Scientific Reports*, 2021, 11.
15. A, W.S.;B, Y.H.A. Global warming over the period 1961–2008 did not increase high-temperature stress but did reduce low-temperature stress in irrigated rice across China - ScienceDirect. *Agricultural and Forest Meteorology*, 2011, 151, 1193-1201.
16. Yin, X.;Olesen, J.E.;Wang, M.;Ozturk, I.;Zhang, H.;Chen, F. Impacts and adaptation of the cropping systems to climate change in the Northeast Farming Region of China. *European Journal of Agronomy*, 2016, 78, 60-72.
17. Cheng, C.;Yun, K.-Y.;Ressom, H.W.;Mohanty, B.;Bajic, V.B.;Jia, Y.;Yun, S.J.;de los Reyes, B.G. An early response regulatory cluster induced by low temperature and hydrogen peroxide in seedlings of chilling-tolerant japonica rice. *Bmc Genomics*, 2007, 8.
18. Jiang, L.;Han, J.;Cui, H.;Chu, Z.;Li, S.;Zhang, Y.;Ji, Y.;Wang, Q.;Li, X.;Wang, P. Effect of Climate Change on Identification of Delayed Chilling Damage of Rice in China's Cold Region. *Agriculture*, 2024, 14, 1456.
19. Oliver, S.N.;Dennis, E.S.;Dolferus, R. ABA regulates apoplastic sugar transport and is a potential signal for cold-induced pollen sterility in rice. *Plant and Cell Physiology*, 2007, 48, 1319-1330.
20. Zhu, J.-K. Abiotic stress signaling and responses in plants. *Cell*, 2016, 167, 313-324.
21. Boyer, J.S. Plant productivity and environmental. *Science*, 1982, 218, 443-448.
22. Shi, Y.;Guo, E.;Cheng, X.;Wang, L.;Jiang, S.;Yang, X.;Ma, H.;Zhang, T.;Li, T.;Yang, X. Effects of chilling at different growth stages on rice photosynthesis, plant growth, and yield. *Environmental and Experimental Botany*, 2022, 203.
23. Allen, D.J.;Ort, D.R. Impacts of chilling temperatures on photosynthesis in warm-climate plants. *Trends in Plant Science*, 2001, 6, 36-42.
24. Paul, M.J.;Foyer, C.H. Sink regulation of photosynthesis. *Journal of Experimental Botany*, 2001, 52, 1383-1400.
25. Subedi, K.D.;Gregory, P.J.;Summerfield, R.J.;Gooding, M.J. Cold temperatures and boron deficiency caused grain set failure in spring wheat (Triticum aestivum L). *Field Crops Research*, 1998, 57, 277-288.
26. Schulz, E.;Tohge, T.;Zuther, E.;Fernie, A.R.;Hincha, D.K. Flavonoids are determinants of freezing tolerance and cold acclimation in Arabidopsis thaliana. *Scientific Reports*, 2016, 6.
27. Yoshida, S.;Uemura, M. Alterations of plasma membranes related to cold acclimation of plants; in Low Temperature Stress Physiology in Crops, P.H. Li,eds., CRC Press: Boca Raton, 2018, 1-203.
28. Hajihashemi, S.;Brestic, M.;Landi, M.;Skalicky, M. Resistance of fritillaria imperialis to freezing stress through gene expression, osmotic adjustment and antioxidants. *Scientific Reports*, 2020, 10.
29. Rezaie, R.;Mandoulakani, B.A.;Fattahi, M. Cold stress changes antioxidant defense system,

phenylpropanoid contents and expression of genes involved in their biosynthesis in Ocimum basilicum L. *Scientific Reports*, 2020, 10.

30. Verbruggen, N.;Hermans, C. Proline accumulation in plants: a review. *Amino Acids*, 2008, 35, 753-759.
31. Xin, Z.G.;Browse, J. Eskimo1 mutants of Arabidopsis are constitutively freezing-tolerant. *Proceedings of the National Academy of Sciences of the United States of America*, 1998, 95, 7799-7804.
32. Nanjo, T.;Fujita, M.;Seki, M.;Kato, T.;Tabata, S.;Shinozaki, K. Toxicity of free proline revealed in an Arabidopsis T-DNA-tagged mutant deficient in proline dehydrogenase. *Plant and Cell Physiology*, 2003, 44, 541-548.
33. Shao, H.;Guo, Q.;Chu, L.;Zhao, X.;Su, Z.;Hu, Y.;Cheng, J. Understanding molecular mechanism of higher plant plasticity under abiotic stress. *Colloids and Surfaces B-Biointerfaces*, 2007, 54, 37-45.
34. Huener, N.P.A.;Bode, R.;Dahal, K.;Hollis, L.;Rosso, D.;Krol, M.;Ivanov, A.G. Chloroplast redox imbalance governs phenotypic plasticity: the "grand design of photosynthesis" revisited. *Frontiers in Plant Science*, 2012, 3.
35. Santarius, K.A. Freezing of isolated thylakoid membranes in complex media.VI. The effect of pH. *Cryobiology*, 1990, 27, 547-561.
36. Hou, W.;Sun, A.H.;Chen, H.L.;Yang, F.S.;Pan, J.L.;Guan, M.Y. Effects of chilling and high temperatures on photosynthesis and chlorophyll fluorescence in leaves of watermelon seedlings. *Biologia Plantarum*, 2016, 60, 148-154.
37. Xu, J.-Z.;Peng, S.-Z.;Yang, S.-H.;Yu, Y.-M. Coupled model of stomatal conductance-photosynthesis-transpiration for paddy rice under water-saving irrigation. *Archives of Agronomy and Soil Science*, 2014, 60, 163-181.
38. Yu, Q.;Goudriaan, J.;Wang, T.D. Modelling diurnal courses of photosynthesis and transpiration of leaves on the basis of stomatal and non-stomatal responses, including photoinhibition. *Photosynthetica*, 2001, 39, 43-51.
39. Grassi, G.;Magnani, F. Stomatal, mesophyll conductance and biochemical limitations to photosynthesis as affected by drought and leaf ontogeny in ash and oak trees. *Plant Cell and Environment*, 2005, 28, 834-849.
40. Buckley, T.N.;Diaz-Espejo, A. Partitioning changes in photosynthetic rate into contributions from different variables. *Plant Cell and Environment*, 2015, 38, 1200-1211.
41. Najeeb, S.;Mahender, A.;Anandan, A.;Hussain, W.;Li, Z.;Ali, J. Genetics and Breeding of Low-Temperature Stress Tolerance in Rice; in Rice Improvement: Physiological, Molecular Breeding and Genetic Perspectives, J. Ali,Wani, S.H.,eds., Springer International Publishing: Cham, 2021, 221-280.
42. Huang, M.;Cao, J.;Liu, Y.;Zhang, M.;Hu, L.;Xiao, Z.;Chen, J.;Cao, F. Low-temperature stress during the flowering period alters the source-sink relationship and grain quality in field-grown late-season rice. *Journal of Agronomy and Crop Science*, 2021, 207, 833-839.
43. Du, S.;Huang, X.;Cai, Y.;Hao, Y.;Qiu, S.;Liu, L.;Cui, M.;Luo, L. Differential antioxidant compounds and activities in seedlings of two rice cultivars under chilling treatment. *Frontiers in Plant Science*, 2021, 12.
44. Wang, J.;Lin, X.;Sun, Q.;Jena, K.K. Evaluation of Cold Tolerance for Japonica Rice Varieties from Different Country. *Advance Journal of Food Science & Technology*, 2013, 5, 54-56.
45. Ham, T.-H.;Kwon, Y.;Lee, Y.;Choi, J.;Lee, J. Genome-Wide Association Study Reveals the Genetic Basis of Cold Tolerance in Rice at the Seedling Stage. *Agriculture*, 2021, 11, 318.
46. Li, T.G.;Bsvergara, R. Correlation of cold tolerance at different growth stages in rice. *Journal of Integrative Plant Biology*, 1981, 23, 203-207.
47. Shimono, H.;Hasegawa, T.;Moriyama, M.;Fujimura, S.;Nagata, T. Modeling spikelet sterility

induced by low temperature in rice. *Agronomy Journal*, 2005, 97, 1524-1536.

48. Xiao, L.;Liu, L.;Asseng, S.;Xia, Y.;Tang, L.;Liu, B.;Cao, W.;Zhu, Y. Estimating spring frost and its impact on yield across winter wheat in China. *Agricultural and Forest Meteorology*, 2018, 260, 154-164.
49. Sumayya, K.;Rajanna, M.P.;Deepak, C.A.;Shivakumar, K.V.;Denesh, G.R. Evaluation of rice (Oryza sativa L.) genotypes for cold tolerance at seedling stage. *Oryza*, 2020, 57, 43-48.
50. Hasina Khatun, H.K.;Biswas, P.S.;Hwang HungGoo, H.H.;Kim KyungMin, K.K. A quick and simple in-house screening protocol for cold-tolerance at seedling stage in rice. *Plant Breeding and Biotechnology*, 2016, 4, 373-378.
51. Jacobs, B.C.;Pearson, C.J. Growth, development and yield of rice in response to cold temperature. *Journal of Agronomy and Crop Science-Zeitschrift Fur Acker Und Pflanzenbau*, 1999, 182, 79-88.
52. Shimono, H.;Hasegawa, T.;Iwama, K. Response of growth and grain yield to cool water at different growth stages in paddy rice. in Increased lowland rice production in the Mekong Region: Proceedings of an International Workshop of the Australian Centre for International Agricultural Research, Vientiane, Laos 2001.
53. Gunawardena, T.A.;Fukai, S.;Blamey, F.P.C. Low temperature induced spikelet sterility in rice. II. Effects of panicle and root temperatures. *Australian Journal of Agricultural Research*, 2003, 54, 947-956.
54. Satake, T.;Koike, S. Sterility caused by cooling treatment at the flowering stage in rice plants: I. The stage and organ susceptible to cool temperature. *Japanese Journal of Crop Science*, 1983, 52, 207-214.
55. Liu, L.;Xia, Y.;Liu, B.;Chang, C.;Xiao, L.;Shen, J.;Tang, L.;Cao, W.;Zhu, Y. Individual and combined effects of jointing and booting low-temperature stress on wheat yield. *European Journal of Agronomy*, 2020, 113.
56. Shi, P.;Zhu, Y.;Tang, L.;Chen, J.;Sun, T.;Cao, W.;Tian, Y. Differential effects of temperature and duration of heat stress during anthesis and grain filling stages in rice. *Environmental and Experimental Botany*, 2016.
57. Raja, V.;Qadir, S.U.;Alyemeni, M.N.;Ahmad, P. Impact of drought and heat stress individually and in combination on physio-biochemical parameters, antioxidant responses, and gene expression in Solanum lycopersicum. *3 Biotech*, 2020, 10.
58. Guo, L.-F.;Liu, J.-P.;Xu, J.-Q.;Wang, Y.-X.;Zhao, L.;Qian, C. Physiological mechanism of exogenous silicon alleviating drought stress in maize. *Science of Advanced Materials*, 2022, 14, 272-285.
59. Batool, T.;Ali, S.;Seleiman, M.F.;Naveed, N.H.;Ali, A.;Ahmed, K.;Abid, M.;Rizwan, M.;Shahid, M.R.;Alotaibi, M.;Al-Ashkar, I.;Mubushar, M. Plant growth promoting rhizobacteria alleviates drought stress in potato in response to suppressive oxidative stress and antioxidant enzymes activities. *Scientific Reports*, 2020, 10.
60. Song, S.-Y.;Chen, Y.;Chen, J.;Dai, X.-Y.;Zhang, W.-H. Physiological mechanisms underlying OsNAC5-dependent tolerance of rice plants to abiotic stress. *Planta*, 2011, 234, 331-345.
61. Li, H.;Zhu, Y.;Hu, Y.;Han, W.;Gong, H. Beneficial effects of silicon in alleviating salinity stress of tomato seedlings grown under sand culture. *Acta Physiologiae Plantarum*, 2015, 37, 1-9.
62. Xu, J.;Guo, L.;Liu, L. Exogenous silicon alleviates drought stress in maize by improving growth, photosynthetic and antioxidant metabolism. *Environmental and Experimental Botany*, 2022, 201.
63. Bates, L.S.;Waldren, R.P.;Teare, I.D. Rapid determination of free proline for water-stress studies. *Plant and Soil*, 1973, 39, 205-207.
64. Campion, E.M.;Loughran, S.T.;Walls, D. Protein quantitation and analysis of purity. *Methods Mol Biol*, 2011, 681, 229-258.
65. Loutfy, N.;El-Tayeb, M.A.;Hassanen, A.M.;Moustafa, M.F.M.;Sakuma, Y.;Inouhe, M. Changes in the water status and osmotic solute contents in response to drought and salicylic acid treatments in

four different cultivars of wheat (Triticum aestivum). *Journal of Plant Research*, 2012, 125, 173-184.

66. Guo, Z.;Cai, L.;Liu, C.;Chen, Z.;Guan, S.;Ma, W.;Pan, G. Low-temperature stress affects reactive oxygen species, osmotic adjustment substances, and antioxidants in rice (Oryza sativa L.) at the reproductive stage. *Scientific Reports*, 2022, 12.
67. Geng, A.;Wang, X.;Wu, L.;Wang, F.;Wu, Z.;Yang, H.;Chen, Y.;Wen, D.;Liu, X. Silicon improves growth and alleviates oxidative stress in rice seedlings (Oryza sativa L.) by strengthening antioxidant defense and enhancing protein metabolism under arsanilic acid exposure. *Ecotoxicology and Environmental Safety*, 2018, 158, 266-273.
68. Yu, X.-F.;Xian, H.-Y.;Qiu, X.-L.;Zou, B.-L.;Zhang, S.-H.;Liu, G.-S.;Zhang, Y.-F.;Zeng, M.;Zeng, L.-Q.;Yang, S.-M. Coupling effect of low temperature and nitrogen on physiological indexes of rice. *Southwest China Journal of Agricultural Sciences*, 2020, 33, 2190-2197.
69. Zhu, C.-q.;Xu, Q.-s.;Cao, X.-c.;Zhu, L.-f.;Kong, Y.-l.;Jin, Q.-y.;Zhang, J.-h. Effects of substrates with different properties on chilling tolerance of early rice seedlings. *Chinese Journal of Rice Science*, 2021, 35, 503-512.
70. Campos, H.;Trejo, C.;Pena-Valdivia, C.B.;Garcia-Nava, R.;Victor Conde-Martinez, F.;Cruz-Ortega, M.R. Stomatal and non-stomatal limitations of bell pepper (Capsicum annuum L.) plants under water stress and re-watering: Delayed restoration of photosynthesis during recovery. *Environmental and Experimental Botany*, 2014, 98, 56-64.
71. Shimono, H.;Hasegawa, T.;Fujimura, S.;Iwama, K. Responses of leaf photosynthesis and plant water status in rice to low water temperature at different growth stages. *Field Crops Research*, 2004, 89, 71-83.
72. Wang, X.-y.;Yang, X.-g.;Sun, S.;Xie, W.-j. Comparison of potential yield and resource utilization efficiency of main food crops in three provinces of Northeast China under climate change. *Ying yong sheng tai xue bao = The journal of applied ecology*, 2015, 26, 3091-102.
73. Varone, L.;Ribas-Carbo, M.;Cardona, C.;Galle, A.;Medrano, H.;Gratani, L.;Flexas, J. Stomatal and non-stomatal limitations to photosynthesis in seedlings and saplings of Mediterranean species pre-conditioned and aged in nurseries: Different response to water stress. *Environmental and Experimental Botany*, 2012, 75, 235-247.
74. Xingyang, S.;Guangsheng, Z.;Qijing, H.;Huailin, Z. Stomatal limitations to photosynthesis and their critical Water conditions in different growth stages of maize under water stress. *Agricultural Water Management*, 2020, 241.
75. Zeng, Y.;Zhang, Y.;Xiang, J.;Uphoff, N.T.;Pan, X.;Zhu, D. Effects of low temperature stress on spikelet-related parameters during anthesis in indica-japonica hybrid Rice. *Frontiers in Plant Science*, 2017, 8.
76. Albertos, P.;Wagner, K.;Poppenberger, B. Cold stress signalling in female reproductive tissues. *Plant Cell and Environment*, 2019, 42, 846-853.
77. Gao, Y.;Li, Y.;Huang, L.;Zhao, J.;Li, S.;Lu, J.;Li, X.;Yang, T. Identification of the effects of low temperature on grain-setting rate of different types of late-season rice (Oryza sativa L.) during heading. *Field Crops Research*, 2024, 318.
78. Rao, G.-s.;Ashraf, U.;Kong, L.-l.;Mo, Z.-w.;Xiao, L.-z.;Zhong, K.-y.;Rasul, F.;Tang, X.-n. Low soil temperature and drought stress conditions at flowering stage affect physiology and pollen traits of rice. *Journal of Integrative Agriculture*, 2019, 18, 1859-1870.
