# Supplementary material for: Effects of low-temperature stress at different growth stages on rice physiology, pollen viability and yield in China’s cold region
Source: PLoS One. 2025 Aug 13;20(8):e0329441. doi: 10.1371/journal.pone.0329441 (PMC12349717; doi:10.1371/journal.pone.0329441)
Supplement: Attachment — Submitted filename: Response to Reviewers.docx [file pone.0329441.s004.docx]

**Response to Reviewers**

**Dear Editors:**

We are pleased to submit the revised version of our manuscript entitled " **Effects of Low-Temperature Stress at Different Growth Stages on Rice Physiology, Pollen Viability and Yield in China’s Cold Region**" (Manuscript ID: **PONE-D-25-20271**) for consideration in **PLOS ONE**. We sincerely appreciate the time and effort invested by the editorial team and reviewers in evaluating our work. Their constructive comments have significantly enhanced the clarity, rigor, and impact of this paper.

We have carefully addressed all points raised by the reviewers and editors through **comprehensive revisions**. Key modifications include:

- The style and format, such as font, headings, layout and spacing, page numbers, reference style, and other aspects of the manuscript have been revised to meet the standards of the journal.
- In the abstract, the background and significance of the research were elaborated upon, and key quantitative data were incorporated into the presentation of the primary results, thereby enhancing the comprehensiveness of the research conclusion.
- Detailed legends for each figure were rewritten to provide comprehensive descriptions.
- In the "Results" section, a new research focus titled "The correlation between Pn, MDA, and yield/yield-related parameters" has been incorporated.
- At the outset of the "Discussion" section, a paragraph was inserted to further elaborate on the practical significance of the findings of this study in comparison with extant research.

**RESPONSE TO REVIEWERS’ COMMENTS**

**Journal Requirements**

**Comment 1:** Please ensure that your manuscript meets PLOS ONE's style requirements, including those for file naming.

**Response:** We have thoroughly revised the manuscript to ensure full compliance with PLOS ONE's formatting and style requirements. The manuscript structure has been reorganized according to the journal’s guidelines, including the appropriate ordering of sections such as Title, Abstract, Introduction, Materials and Methods, Results, Discussion, Conclusions, Author Contributions, Acknowledgments, and References. Section headings have been standardized to reflect the proper hierarchy and improve clarity. Additionally, all in-text citations and the reference list have been reformatted to follow PLOS ONE's citation style, using numerical order and the appropriate punctuation and spacing conventions.

Figures and tables have been reordered and referenced sequentially within the text, with captions edited for clarity and consistency. Each figure and table now appears in the manuscript after its first mention, in accordance with journal requirements. All submitted files have been renamed to match PLOS ONE’s file naming guidelines (e.g., “Fig1.tif,” “S1_Table.docx”) to facilitate the editorial and publication process.

Furthermore, we have carefully edited the manuscript for language quality, ensuring grammatical accuracy, consistent terminology, and professional academic tone throughout. We appreciate the reviewer’s suggestion and have taken all necessary steps to align the manuscript with the journal’s stylistic and submission standards.

**Comment 2:** Please state what role the funders took in the study.

**Response:** We appreciate the opportunity to clarify the role of the funding sources in this research. Below is a detailed statement addressing the journal's request:

**Funding Statement:** This work was funded by the National Key Research and Development Program of China (No. 2022YFD2300201), the Natural Science Foundation of Heilongjiang Province (No. LH2024D020), the National Natural Science Foundation of China (No. 31671575) and the Joint Foundation on Regional Meteorological S & T Collaborative Innovation of Northeast China (No. 2024GX006).

**The National Key Research and Development Program of China** provided financial support for personnel salaries, experimental materials, and data analysis.

**The Natural Science Foundation of Heilongjiang Province** provided financial support for personnel salaries, experimental materials, and data analysis.

**The National Natural Science Foundation of China** funded equipment access “Hitachi H7650” and publication fees.

**The Joint Foundation on Regional Meteorological S & T Collaborative Innovation of Northeast China** provided financial support for personnel salaries, experimental materials, and data analysis.

The funders played role in:

- Study design
- Data collection and analysis
- Decision to publish
- Preparation of the manuscript

**Comment 3:** We note that your Data Availability Statement is currently as follows: All relevant data are within the manuscript and its Supporting Information files.

**Response:** We appreciate the editor’s observation regarding the phrasing of the Data Availability Statement. Upon review, we acknowledge that the original statement did not accurately reflect the current status of the data. We submitted the experimental analysis data and figures from the manuscript as Supporting Information ZIP files in the form of a compressed package through the submission system. This revision ensures transparency regarding the availability of data while also protecting the integrity and confidentiality of the ongoing research.

**Review Comments to the Author**

**Reviewer #1:**

**Comment:** This is an informative manuscript, and I appreciate the authors’ efforts in the experimental work and data analysis. However, several aspects of the manuscript would benefit from clarification and revision to improve scientific rigor, language clarity, and overall presentation. Please refer to in the manuscript for detailed suggestions.

**Response:** We are grateful to the reviewer for the recognition of the manuscript’s overall contribution and for the thoughtful suggestions aimed at enhancing its scientific rigor, language clarity, and presentation. We fully acknowledge that despite the strength of the experimental design and dataset, the initial version of the manuscript could benefit from further refinement in presentation and clarity. In response, we have conducted a meticulous revision of the entire manuscript based on the detailed suggestions provided. Specifically, we have clarified methodological descriptions, including the experimental design across multiple growth stages, and elaborated on key physiological indicators such as photosynthetic parameters, ROS content, and antioxidant enzyme activity, to improve reproducibility and transparency.

To enhance scientific rigor, we also revised the interpretation of the statistical analyses, clearly reported p-values and significance thresholds, and added clarification on experimental replicates and control conditions. Additionally, we restructured and streamlined sections of the Introduction and Discussion to strengthen logical flow, emphasize novelty, and improve readability. The language throughout the manuscript has been carefully edited to eliminate grammatical ambiguities and ensure precise scientific communication. Furthermore, we have carefully revised all figure legends and ensured that tables and figures are fully self-explanatory and consistent with the text.

Importantly, we have also emphasized the broader scientific relevance of our findings—particularly how our dynamic, growth-stage-specific LTS assessments under natural-like simulation conditions provide practical implications for field-level identification of cold stress in rice, which is often lacking in previous studies. We believe that the revised manuscript better reflects the quality and depth of the research and presents our findings in a clearer and more accessible manner. We are sincerely grateful to the reviewer for the comments, which have substantially helped us improve the overall quality of our work.

**Reviewer #2:**

**Comment 1:** Insufficient mechanistic depth in key findings such as non-stomatal limitation and pollen sterility.

**Response:** We sincerely thank the reviewer for highlighting the need to strengthen the mechanistic explanation of key findings, particularly regarding non-stomatal limitations of photosynthesis and pollen sterility under low-temperature stress (LTS). We fully agree that enhancing the depth of interpretation in these areas is essential to improve the scientific impact of the manuscript. In response, we have added detailed mechanistic discussions supported by recent literature and our experimental evidence. Specifically, we expanded our explanation of non-stomatal limitations by linking observed reductions in Pn with ultrastructural damage to chloroplasts and impaired biochemical processes such as Rubisco activity, emphasizing that such limitations are not merely physiological responses but are underpinned by cellular structural disruptions (page 19, lines 1083–1085). Furthermore, we elaborated on the mechanisms of pollen sterility by discussing how LTS interferes with anther development, tapetal degradation, and pollen wall formation—key biological processes that are particularly vulnerable during the booting and heading stages (pages 24–25, lines 1337–1358). These additions provide a clearer and more comprehensive understanding of the physiological and cytological basis of the observed phenotypic changes. We hope these revisions effectively address the reviewer’s concern and contribute to a more mechanistically robust manuscript.

**Comment 2:** No quantitative definition of stress thresholds.

**Response:** We appreciate the reviewer’s valuable observation regarding the use of the term “stress thresholds.” Upon review, we agree that the term may have caused confusion by implying a specific quantitative threshold, which was not the original intent. In our study, the objective was to qualitatively describe the dynamic response patterns of physiological and biochemical indicators under low-temperature stress (LTS), rather than to define a precise quantitative threshold value. Our findings showed that key indicators—such as antioxidant enzyme activities (SOD, POD, CAT) and osmolyte contents (proline, soluble sugars, and proteins)—tended to increase initially with the duration of LTS and then decline, typically peaking at day 7 (D7). This pattern reflects a time-dependent stress adaptation process, rather than a clearly defined physiological threshold. To avoid misinterpretation, we have revised the wording throughout the manuscript to more accurately convey this trend. For instance, we now use expressions such as “showed a trend of initially increasing and subsequently decreasing” (page 2, lines 83–84) to better reflect the qualitative nature of these observations. We thank the reviewer for drawing attention to this point, which allowed us to refine our terminology and improve the scientific precision of the manuscript.

**Comment 3:** Absence of yield-physiology correlation analysis.

**Response:** We thank the reviewer for pointing out the absence of a yield-physiology correlation analysis, which is indeed a critical aspect for understanding the functional relationship between physiological responses and agronomic outcomes under low-temperature stress (LTS). In response, we have added a comprehensive correlation analysis section to the manuscript (pages 21–22, lines 1240–1271). This new analysis includes Pearson correlation coefficients between key physiological parameters—such as net photosynthetic rate (Pn) and malondialdehyde (MDA)—and yield components, including grain yield, effective panicles, seed setting percentage, and 1000-grain weight. Our results revealed a significant positive correlation between Pn and grain yield, as well as a strong negative correlation between MDA and yield-related traits. These findings provide robust evidence that physiological damage caused by LTS, particularly oxidative stress and reduced photosynthetic efficiency, directly contributes to yield losses. By including this analysis, we believe the manuscript now more clearly illustrates the mechanistic link between physiological responses and final agronomic performance, thereby strengthening the overall scientific rigor and practical relevance of the study. We appreciate the reviewer’s suggestion, which has significantly improved the clarity and depth of our findings.

**Comment 4:** Substantial language editing needed throughout the manuscript. Moreover, the manuscript would benefit from expanded discussion on practical implications and comparison with other studies.

**Response:** Thank you for your valuable feedback, particularly regarding the need for substantial language editing and the importance of strengthening the discussion with practical implications and comparisons to existing studies. We fully agree with these observations and have taken concrete steps to address them. First, we conducted a thorough, line-by-line language revision of the entire manuscript to improve clarity, precision, and fluency. This included correcting grammatical errors, improving sentence structure, and enhancing the overall readability of the text to ensure it meets the standards of international academic publishing.

Second, we have expanded the Discussion section to better contextualize our findings in light of previous research and to highlight their practical significance. Specifically, we elaborated on the implications of our results for cold-tolerant rice breeding, stress monitoring in field conditions, and future climate resilience strategies (pages 22–23, lines 1273–1291). We also compared our findings with prior studies on low-temperature stress in rice, emphasizing the novelty of our dynamic simulation approach and the identification of growth-stage-specific vulnerability. These additions not only strengthen the scientific depth of the manuscript but also underscore its relevance for agronomists, breeders, and policymakers. We appreciate the reviewer’s suggestions, which helped us substantially improve the scientific and communicative quality of the manuscript.

**General Comments**

**The minor points**

**Abstract**:

**Comment 1:** The abstract should accurately reflect the full scope of the study, including the background, objectives, methods, key results (with quantitative data where possible), and main conclusions or novelty.

Response:

We sincerely thank the reviewer for this valuable suggestion. We fully agree that the abstract plays a critical role in clearly and concisely conveying the full scope of the study. In response, we have carefully revised the abstract to include all essential components: a concise background establishing the importance of the study, clearly stated objectives, a summary of the methodology (including the experimental design across tillering, booting, and heading stages under controlled low-temperature stress conditions), and key findings supported by specific quantitative results. For instance, we now explicitly report the reductions in net photosynthetic rate (Pn), chloroplast damage, antioxidant activity dynamics, and yield losses at different growth stages, providing clear numerical ranges. We have also strengthened the concluding sentences to better highlight the novelty and practical implications of our findings, particularly the stage-specific sensitivity of rice to LTS and its relevance for cold-tolerance breeding and climate-resilient cultivation strategies. We believe these revisions significantly improve the clarity, scientific value, and completeness of the abstract, and we appreciate the reviewer’s guidance in prompting these important improvements.

**Comment 2:** All abbreviations should be spelled out in full when first mentioned in the abstract, in accordance with journal style. Please ensure consistency throughout the manuscript as well.

Response:

Thank you for this important observation. In accordance with PLOS ONE’s style requirements, we have carefully reviewed the entire manuscript, including the abstract, to ensure that all abbreviations are fully spelled out upon first mention. For example, terms such as “low-temperature stress (LTS),” “photosynthetic rate (Pn),” and “superoxide dismutase (SOD)” are now clearly defined when first introduced. We have also ensured that each abbreviation is used consistently thereafter throughout the text, tables, and figure legends. This revision improves clarity and accessibility for a broad readership and aligns the manuscript with the journal’s formatting standards. We appreciate the reviewer’s guidance in helping us strengthen the manuscript’s presentation.

**Comment 3:** It is recommended that key quantitative results be explicitly stated in the abstract—for example, the percentage reduction in net photosynthetic rate (P_n_), yield loss, and pollen viability decline. This will enhance the informativeness and scientific rigor of the abstract.

Response:

We thank the reviewer for this constructive recommendation. We fully agree that including key quantitative results in the abstract enhances its clarity, informativeness, and scientific rigor. In response, we have revised the abstract to explicitly state representative quantitative findings from our study. Specifically, we now include the percentage reductions in net photosynthetic rate (Pn), which reached up to 93.9% at the booting stage under severe LTS; grain yield losses, which ranged from 21.62% to 88.76% depending on the growth stage and stress duration; and pollen viability decline, which was most pronounced at the heading stage with a reduction of 44.67%. These revisions are located on page 1, lines 21–26, and further emphasized on page 2, lines 84–87. We believe these additions significantly strengthen the scientific impact of the abstract by clearly conveying the magnitude of physiological and agronomic responses to LTS. We appreciate the reviewer’s suggestion, which helped us improve the precision and value of our summary.

**Comment 4:** The term "non-stomatal limiting factor" is somewhat vague. Please clarify whether it refers to inhibition of photosynthetic enzymes, damage to chloroplast ultrastructure, or other specific mechanisms.

Response:

We appreciate the reviewer’s thoughtful comment regarding the term “non-stomatal limiting factor.” We agree that this phrase, while commonly used, can be imprecise without further clarification. To improve clarity, we have removed the term from the abstract to avoid ambiguity. In the Discussion section, we now explicitly elaborate on the underlying mechanisms contributing to the decline in photosynthetic capacity under prolonged and intense low-temperature stress (LTS). Based on our physiological measurements and supported by previous literature, we interpret the observed reduction in net photosynthetic rate (Pn) as being primarily caused by non-stomatal limitations—specifically, damage to the chloroplast ultrastructure, impaired thylakoid membrane integrity, and potential inhibition of enzymatic activity involved in carbon fixation (e.g., Rubisco). These structural and biochemical disruptions reduce the efficiency of CO₂ assimilation despite adequate stomatal conductance. This interpretation is supported by our transmission electron microscopy observations and the elevated levels of oxidative stress markers. We believe this clarification strengthens the mechanistic interpretation of our findings and aligns with the reviewer’s request for greater specificity.

**Comment 5:** The abstract appears to report data from one experimental year, while the main text presents two years of data. Please verify and revise for consistency.

Response:

We sincerely thank the reviewer for bringing this to our attention. We apologize for the inconsistency between the abstract and the main text regarding the duration of the experimental study. We confirm that all findings presented in this manuscript are based on data collected over two consecutive years (2023 and 2024), as described in the Materials and Methods section. To ensure accuracy and consistency, we have revised the abstract to clearly state that the study was conducted over a two-year period using a temperature-controlled field experiment. This correction ensures alignment between the abstract and the body of the manuscript and reflects the robustness and reproducibility of our results. We appreciate the reviewer’s attention to detail, which helped us improve the clarity and accuracy of our work.

**Comment 6:** Consider adding a closing sentence to the abstract that summarizes the practical implications of the findings, such as their relevance to cold stress modeling, rice cultivation strategies, or breeding for cold tolerance.

Response:

We thank the reviewer for this helpful suggestion. We agree that concluding the abstract with a statement highlighting the practical implications of our findings enhances the relevance and impact of the study. In response, we have added a final sentence to the abstract (page 2, lines 87–89) that emphasizes the applicability of our results to real-world agricultural challenges. Specifically, the revised abstract now notes how our findings provide a theoretical basis for identifying and evaluating low-temperature stress in rice under field conditions, and offer a methodological reference for the monitoring of cold stress in other crops. This addition underscores the broader significance of the research for cold stress modeling, climate-resilient cultivation strategies, and the development of cold-tolerant rice varieties. We believe this strengthens the abstract by clearly linking the experimental outcomes to their practical applications.

**Introduction:**

**Comment 1:** Please consider revising the opening sentence of the Introduction by changing “The rice plant” to “Rice”, which is more concise and conventional in scientific writing. In addition, the manuscript would benefit from thorough English language editing to improve clarity, grammar, and readability.

Response:

We thank the reviewer for this valuable suggestion. In response, we have revised the opening sentence of the Introduction by replacing “the rice plant” with the more concise and conventional term “rice” (page 2, line 93), in accordance with standard scientific writing conventions. Furthermore, we have carefully reviewed the entire manuscript and conducted a comprehensive English language edit to improve clarity, grammar, and overall readability. This includes refining sentence structures, eliminating redundancy, and enhancing the precision of technical terms. We believe these revisions have substantially improved the linguistic quality and accessibility of the manuscript. We sincerely appreciate the reviewer’s recommendation, which helped us strengthen both the presentation and professionalism of our work.

**Comment 2:** Expand the literature review to include recent insights into cold stress responses in rice, especially on hormonal signaling (e.g., ABA, JA, SA) and regulatory networks.

Response:

We appreciate the reviewer’s insightful suggestion regarding the expansion of the literature review to include recent advances in understanding cold stress responses in rice, particularly related to hormonal signaling and regulatory mechanisms. In response, we have revised the Introduction to incorporate an expanded discussion on the roles of key plant hormones—abscisic acid (ABA), jasmonic acid (JA), and salicylic acid (SA)—in mediating cold stress responses (page 3, lines 218–224). This section now highlights how these hormones regulate physiological and molecular processes such as stomatal closure, reactive oxygen species (ROS) homeostasis, gene expression, and stress signaling cascades, thereby contributing to cold tolerance. By integrating recent findings in this area, we aim to provide a more comprehensive foundation for the study and to better contextualize our own results within current research. We thank the reviewer for this valuable recommendation, which has significantly improved the depth and relevance of the manuscript’s theoretical background.

**Comment 3:** Introduce more clearly the cold tolerance background of the two rice cultivar.

Response:

We thank the reviewer for highlighting the need to provide a clearer introduction to the cold tolerance background of the rice cultivar used in this study. To address this, we have clarified early in the manuscript that this study specifically focused on the japonica rice variety Longgeng 31, which is widely cultivated in the cold-region province of Heilongjiang, China. We have elaborated on its agronomic importance and its moderate cold tolerance in the context of regional climate challenges and breeding programs. This additional context is now presented more explicitly in the manuscript (lines 1261–1263), ensuring that readers can better understand the rationale for selecting this cultivar and its relevance to the objectives of our research. We appreciate the reviewer’s suggestion, which helped us improve the clarity and completeness of our experimental background.

**Comment 4:** Explicitly state the scientific novelty of this study compared to existing research.

Response:

We thank the reviewer for this important comment. In response, we have explicitly stated the scientific novelty of our study in the Introduction section (lines 276–287). Unlike previous research that primarily focused on constant or artificially simplified low-temperature stress conditions at isolated growth stages, our study employed a dynamic, hourly variable temperature simulation that more accurately mimics natural field conditions. Furthermore, we comprehensively evaluated physiological, biochemical, and reproductive responses to low-temperature stress across three critical rice growth stages—tillering, booting, and heading—over two consecutive years. This integrated, multi-dimensional approach enabled us to identify stage-specific sensitivity to cold stress, reveal key physiological mechanisms such as non-stomatal limitations and antioxidant responses, and establish correlations with yield-related outcomes. These aspects represent a significant advancement over existing studies and contribute new insights into cold tolerance evaluation in rice under near-realistic environmental conditions. We appreciate the reviewer’s suggestion, which prompted us to better highlight the unique contributions of our work.

**Comment 5:** Strengthen the articulation of the study‘s aims by emphasizing its relevance to rice cold stress physiology and breeding.

Response:

We appreciate the reviewer’s suggestion to more clearly articulate the aims of the study and emphasize their relevance to rice cold stress physiology and breeding. In response, we have revised the Introduction to explicitly highlight the objectives of our research within the broader context of understanding physiological mechanisms and supporting the development of cold-tolerant rice cultivars. As detailed in lines 287 to 295, we now clearly state that this study aims to (1) elucidate the physiological and biochemical responses of rice to low-temperature stress at different developmental stages, (2) identify the most sensitive growth stages to cold stress, and (3) provide a theoretical foundation and practical reference for cold-tolerant rice breeding programs and climate-resilient cultivation strategies. These additions improve the clarity of the study’s purpose and underline its significance for advancing both scientific understanding and agricultural application. We thank the reviewer for this insightful recommendation.

**Materials and methods:**

**Comment 1:** Lack of replication details: The number of biological replicates and technical replicates per treatment is not clearly stated. For example, were measurements such as Pn or enzyme activity based on three or four replicates? Please specify the replication scheme for each experimental component.

**Response:** We thank the reviewer for pointing out the need to clarify the replication scheme used in our experiments. In response, we confirm that all physiological, biochemical, and yield-related measurements—including parameters such as net photosynthetic rate (Pn), enzyme activity, and reactive oxygen species content—were based on three independent biological replicates per treatment, each with corresponding technical replicates to ensure accuracy and reproducibility. To improve transparency and consistency, we have now explicitly stated the number of replicates in the figure captions and relevant sections of the Methods. These revisions ensure that readers and reviewers can fully understand the experimental design and the statistical basis of our results. We appreciate the reviewer’s observation, which helped us enhance the methodological clarity of the manuscript.

**Comment 2:** Incomplete description of greenhouse temperature control: It remains unclear whether the temperature treatments were constant or simulated by daily temperature fluctuations to reflect natural field conditions (i.e., diurnal variation vs. fixed temperatures). Does Figure 1 show actual temperature fluctuation curves? This should be clearly described in the text.

Response:
We appreciate the reviewer’s valuable comment regarding the clarity of our temperature treatment protocol. To simulate realistic field conditions, our low-temperature stress (LTS) treatments were conducted under a 24-hour variable temperature regime rather than fixed or constant temperatures. These diurnal temperature cycles were specifically designed to mimic the natural fluctuations observed in cold-prone rice cultivation regions, particularly in northeast China. Each LTS treatment followed a pre-programmed hourly temperature profile that reflects field-like day–night changes in ambient temperature, rather than maintaining a static setting. The actual hourly fluctuation curves are presented in Figure 1, which accurately depicts the temporal thermal dynamics used in the phytotron.

We have now explicitly described this setup in the revised Materials and Methods section to avoid ambiguity. This dynamic simulation represents a methodological advancement over many previous studies that applied constant low-temperature treatments, which may oversimplify stress exposure. Our approach aligns with recent recommendations in cold stress research to incorporate diurnal rhythms in stress simulations for improved ecological relevance (e.g., Shi et al., 2022, Environmental and Experimental Botany; Shimono et al., 2007, Field Crops Research). We believe this clarification enhances the reproducibility and physiological relevance of our study design.

**Comment 3:** Missing agronomic management details: Important background parameters are lacking, such as fertilizer application rates, irrigation methods, pest and disease control measures, and whether such factors were standardized across treatments. These are essential for reproducibility and should be included.

Response:
Thank you for pointing out the need for more comprehensive agronomic context. We have now included detailed information on agronomic management practices in the Materials and Methods section (lines 329–334). All rice pots received uniform baseline management to ensure consistency across treatments and to isolate temperature as the primary experimental variable. Specifically:

Fertilization: Each pot was treated with 2 g ammonium sulfate, 1 g potassium sulfate, and 1 g diammonium phosphate as basal fertilizers, with an additional 1 g ammonium sulfate applied during the panicle initiation stage.

Irrigation: Continuous flooding was maintained throughout the experiment, except for a short period during the late tillering stage to encourage effective tiller formation.

Weed and Pest Control: Weeds were manually removed, and standard pesticide applications were applied uniformly across all treatments to manage pests and diseases.

These measures followed local agronomic standards and were uniformly implemented across all treatment groups. By standardizing these environmental and management factors, we ensured that any observed physiological or agronomic differences were attributable to LTS treatments alone. This level of control strengthens the internal validity and reproducibility of our study.

**Comment 4**: The Materials and Methods section (Page 3, Line 135) cited references incorrectly. The citation of reference (No. 15) needs to be removed.

Response:
We thank the reviewer for identifying this citation error. The incorrect citation of reference No. 15 has been removed from the relevant section, and we have cross-checked all remaining citations for accuracy and consistency.

**Comment 5:** The Materials and Methods section (Page 5, Line 188) mentioned the yield and its composition. However, the method for calculating the yield composition should be provided.

Response:
We agree with the reviewer that the calculation of yield components must be clearly defined. In response, we have added a reference in line 383 that details the standardized method used for calculating grain yield and its components, including 1000-grain weight, grain number per panicle, effective panicles per pot, and seed setting percentage. This reference (Campion et al., 2018) is widely cited in crop physiology studies and provides clear methodological guidance to ensure reproducibility and standardization.

**Comment 6:** What is the basis for choosing Longgeng31 as the experimental variety? How is the cultivation situation of this variety in the cold regions of China?

Response:
We appreciate the reviewer’s interest in the rationale for cultivar selection. As clarified in the revised manuscript (lines 1354–1356), Longgeng31 was chosen as the test variety due to its regional importance and moderate cold tolerance. It is a japonica cultivar widely grown in Heilongjiang Province, the largest and coldest rice-producing area in China. Longgeng31 has been a staple variety in this region for over two decades due to its stable yield performance, adaptability to low temperatures, and high economic value. Its relevance to real-world production systems makes it an ideal candidate for studying cold stress mechanisms and for providing breeding-oriented insights. Using this representative variety ensures that the outcomes of our study are both scientifically valid and agronomically meaningful.

**Comment 7:** In line 192, "hr" should be changed to "h". Please carefully check the spelling of the entire text.

Response:
We thank the reviewer for noting this detail. The abbreviation “hr” has been corrected to “h” in line with SI unit conventions. In addition, we have performed a careful proofreading of the entire manuscript to ensure consistency in spelling, terminology, and formatting throughout the text.

**Comment 8:** The preparation of chloroplast electron microscope samples requires supplementary references.

Response:
We agree that appropriate referencing is necessary for reproducibility. In the revised manuscript (lines 472–473), we have added a methodological reference for chloroplast sample preparation for transmission electron microscopy (TEM). The protocol follows widely accepted standards in plant ultrastructural analysis and is adapted from Li et al. (2021) and Song et al. (2022), which provide detailed procedures for fixation, embedding, and imaging of chloroplast structures. This addition ensures methodological transparency and supports replication by other researchers.

**Results:**

**Comment 1**: Inconsistent significance annotations in figures.

The use of lettering (e.g., a/b/c) to indicate significant differences is inconsistent and at times confusing across multiple treatment groups. Please standardize the notation and provide clear explanations in the figure legends.
Response:
We thank the reviewer for this important observation. We have carefully reviewed and revised all figures to ensure that the significance annotations are applied consistently across treatment groups. Specifically, we have adopted the use of lowercase letters (e.g., a, b, c) to denote statistically significant differences among treatments within each figure, based on one-way ANOVA followed by LSD post hoc tests at P < 0.05. A standard explanation has been added to each relevant figure legend: “Different lowercase letters indicate significant differences between treatments at P < 0.05.” These revisions improve the interpretability and consistency of the graphical results.

**Comment 2**: Lack of integrative data analysis.

The results section would benefit from additional correlation or regression analyses, such as between Pn and yield, or MDA content and seed setting rate. These would strengthen the biological relevance of the findings.
Response:
We appreciate the reviewer’s insightful suggestion to enhance the integrative analysis of physiological and agronomic traits. In response, we have added a dedicated subsection titled “The correlation between Pn, MDA, and yield-related parameters” (lines 1313–1344), in which we present Pearson correlation analyses among key physiological indicators (Pn and MDA) and yield components (grain yield, seed setting rate, effective panicle number, and 1000-grain weight). The results show a positive correlation between Pn and yield traits, and a strong negative correlation between MDA and yield, thereby reinforcing the biological interpretation of stress-induced yield reductions. These new analyses substantiate the mechanistic connections between stress physiology and agronomic outcomes, enhancing the overall impact of the findings.

**Comment 3**: Limited physiological interpretation of differences.

The manuscript often states that differences are “significant” without discussing their physiological implications. Please elaborate on what these differences suggest in terms of stress response or metabolic disruption.
Response:
We thank the reviewer for highlighting this important point. To address this, we have expanded the discussion of physiological implications in the new section “The correlation between P_n_, MDA, and yield-related parameters” (lines 1313–1344). In this section, we interpret significant changes in photosynthetic rate (P_n_) as indicative of non-stomatal biochemical inhibition under LTS, and elevated MDA as a marker of oxidative membrane damage. We further explain how these changes reflect metabolic stress and contribute to impaired pollen viability and reduced yield. These insights clarify the physiological relevance of the observed differences and support a more mechanistic understanding of cold stress responses in rice.

**Comment 4:** Lack of a quantitative threshold for “critical stress.”

The manuscript mentions a “critical threshold” at 7 days of LTS, but the physiological basis or criteria for this threshold (e.g., ROS peak, Pn decline percentage) is not well-defined. Please include a quantitative definition.
Response:
We appreciate the reviewer’s feedback and recognize that the phrase “critical stress threshold” may have been misleading. In this study, our intention was not to define a strict physiological threshold but rather to describe the temporal pattern of stress response. We observed that several physiological and biochemical parameters—including antioxidant enzyme activity and osmolyte content—peaked around day 7 (D7) of LTS exposure before declining, indicating a turning point in the stress response. To avoid confusion, we have revised the relevant expressions in the manuscript to state that these indicators “showed a trend of initially increasing and subsequently decreasing” (page 2, lines 83–84), which more accurately reflects the observed dynamics without implying a defined threshold value.

**Comment 5:** Language and grammar issues.

Some terms are grammatically incorrect or colloquial, such as “memorably increased” or “spanking reduced.” These should be replaced with scientifically appropriate expressions like “significantly increased” or “markedly decreased.”
Response:
We sincerely thank the reviewer for noting these language issues. We have carefully revised the manuscript for grammar, terminology, and scientific tone. All informal or imprecise expressions—such as “memorably increased” and “spanking reduced”—have been corrected to scientifically appropriate terms, including “significantly increased,” “markedly decreased,” and “notably elevated.” In addition, a full language check has been conducted across the manuscript to ensure clarity, accuracy, and consistency throughout.

**Comment 6:** Chloroplast ultrastructure results lack quantification.

While transmission electron microscopy (TEM) images are useful, there is no quantification of structural changes (e.g., number of grana stacks, chloroplast area, membrane disorganization). Consider including image-based quantification to strengthen this section.
Response:
We thank the reviewer for this suggestion. In this study, our aim was to qualitatively assess chloroplast ultrastructural damage under LTS using representative TEM images from the booting stage. The focus was on comparing structural integrity across treatment conditions (CK, T1, T3) to visualize chloroplast distortion, grana disorganization, and thylakoid damage. As we did not perform image-based quantitative morphometric analysis (e.g., chloroplast area or grana count), we have revised the manuscript to clearly state that this section presents qualitative observations, supported by consistent visual trends across multiple fields of view. These observations complement our physiological data and collectively illustrate how LTS impairs chloroplast function, thus reducing photosynthetic performance. We agree that future studies could benefit from adding quantitative metrics and acknowledge this as a potential follow-up.

**Comment 7:** Supplement the legend of Figure 16.

Response:
Thank you for your helpful suggestion. We have revised and expanded the legend of Figure 16 to clearly describe the measurement of pollen viability, the staining method used (I₂-KI solution), the definition of viability, and the meaning of error bars and significance letters. This revision ensures that the figure is self-contained and interpretable without reference to the main text.

**Discussion:**

**Comment 1:** Insufficient mechanistic interpretation: The discussion lacks depth regarding the mechanisms of non-stomatal limitations. Please consider whether reduced photosynthesis is related to decreased Rubisco activity, PSII inhibition, or impaired ATP synthesis, and support the interpretation with relevant literature.

Response:
We appreciate the reviewer’s important observation regarding the need for deeper mechanistic analysis of the observed photosynthetic inhibition. In the revised discussion (page 25, lines 1408–1419), we reexamined our data in the context of current literature to clarify whether the reduction in net photosynthetic rate (Pn) under long-term LTS was driven primarily by non-stomatal factors. Our findings—particularly the sustained reduction in stomatal conductance (Gs) and parallel decline in Pn—suggest that stomatal limitation was the dominant factor under prolonged stress. However, we also acknowledge that severe LTS could potentially disrupt the internal photosynthetic apparatus.

To address this, we incorporated a discussion referencing relevant studies (e.g., Yamori et al., 2014; Allen & Ort, 2001), which highlight that long-term chilling can inhibit Rubisco activity, damage photosystem II (PSII), and impair ATP synthesis by destabilizing thylakoid membranes. While we did not directly measure Rubisco activity or chlorophyll fluorescence in this study, the evidence from ultrastructural chloroplast damage and elevated MDA levels under severe stress supports the possibility of partial non-stomatal inhibition at the biochemical level. We now clarify that while stomatal closure played a major role, non-stomatal limitations such as PSII impairment and chloroplast dysfunction may also contribute under high-intensity LTS, especially during the booting and heading stages.

**Comment 2:** Limited discussion on pollen viability reduction: The manuscript does not adequately explore the physiological basis of reduced pollen viability. It is recommended to discuss potential disruptions in nutrient transport, anther development, or hormonal regulation during cold stress.

Response:
We thank the reviewer for pointing out the need to expand the physiological interpretation of pollen viability loss under LTS. In the revised manuscript (page 27, lines 1539–1547), we have added a detailed discussion on the factors contributing to reduced pollen viability. Specifically, we discuss how cold stress impairs anther dehiscence, delays tapetal degeneration, and disrupts carbohydrate and nutrient transport to pollen grains, all of which are essential for viable pollen development. We also highlight the role of hormonal imbalance, such as reduced gibberellin and auxin levels under low temperature, in inhibiting reproductive organ development. These mechanisms are supported by recent studies (e.g., Jagadish et al., 2010; Zhang et al., 2017) and help explain the substantial decline in seed setting rate and fertility observed during the booting and heading stages in our study.

**Comment 3:** Lack of comparison with existing studies: The discussion would benefit from comparisons with related research on japonica rice in cold regions, indica varieties, or known cold-tolerant cultivars, in order to contextualize the findings within the broader scientific literature.

Response:
We appreciate this recommendation and have addressed it by adding comparisons with related studies in the early part of the Discussion section (page 23, lines 1346–1358). We now contrast our findings with previous research on japonica rice cultivars from cold regions, as well as on known cold-sensitive indica varieties. Our results align with reports showing greater cold sensitivity at the reproductive stage, but our approach—using dynamic, diurnally variable LTS—adds ecological relevance and reveals more precise stage-specific physiological responses. We also reference studies identifying QTLs for cold tolerance and relate our observations to the physiological traits often associated with those loci (e.g., high antioxidant capacity and membrane stability). These additions strengthen the manuscript by placing our work in the context of current genetic and physiological research on cold stress tolerance.

**Comment 4:** Missing implications for agricultural practices: Consider adding a paragraph that summarizes the practical significance of the findings. This could include recommendations for field management under LTS (e.g., adjustment of sowing dates, fertilization strategies, water layer control), or suggest screening indicators for breeding cold-tolerant japonica rice.

Response:
We thank the reviewer for encouraging us to emphasize the applied relevance of our research. In response, we have added a dedicated paragraph on the agronomic implications of our findings in the first part of the Discussion section (pages 23–24, lines 1358–1363). We highlight how our identification of D7 as a physiological turning point provides practical guidance for early detection of LTS injury and for optimizing sowing dates to avoid sensitive stages overlapping with expected cold spells. Additionally, our results suggest that photosynthetic efficiency and ROS-related parameters could serve as reliable screening indicators for breeding programs targeting cold-tolerant japonica rice. We also briefly discuss how water management and nutrient supplementation could potentially mitigate cold stress during reproductive stages. These recommendations help translate our experimental results into actionable insights for both breeders and farmers.

**Comment 5:** Why are the booting and heading stages more sensitive to LTS? What are the differences in the physiological characteristics of rice at these two stages?

Response:
Thank you for this insightful question. In our revised discussion, we emphasize that booting and heading stages are critical periods for pollen development, spikelet formation, and reproductive organ differentiation, all of which are highly susceptible to low temperatures. Compared to the tillering stage, these later stages exhibit higher metabolic demands, increased hormonal sensitivity, and reduced physiological plasticity. LTS during booting interferes with tapetum degeneration and pollen wall formation, while cold stress during heading disrupts pollen release and fertilization. These vulnerabilities lead to greater reductions in pollen viability and seed setting rate, as observed in our study. Moreover, the impaired photosynthetic activity and chloroplast ultrastructure damage during these stages further compromise assimilate supply to the developing panicle, explaining the more severe yield penalties at booting and heading compared to tillering.

**Comment 6:** The discussion section (Page 17, Paragraph 1) focuses on the alterations in the ultrastructure of rice chloroplasts under LTS. A comparison with existing studies is needed.

Response:
We appreciate the reviewer’s recommendation and have now added a comprehensive comparison with existing studies on chloroplast ultrastructure under cold stress in rice and other cereal crops (lines 1420–1484). Our observations of swollen chloroplasts, disrupted grana stacking, and disintegrated thylakoid membranes are consistent with findings reported by Wang et al. (2016) and Zhao et al. (2019), which demonstrated that LTS leads to plastid instability and reduced photochemical efficiency. Additionally, we discuss how cold-induced oxidative stress contributes to membrane lipid peroxidation, further aggravating chloroplast disintegration. These structural disruptions offer a visual confirmation of the physiological stress markers observed in our study, reinforcing the conclusion that LTS compromises photosynthetic capacity through both biochemical and cellular pathways.

**Closing Statement**

We extend our deepest gratitude to you and the reviewers for your thorough and constructive evaluation of our manuscript. The insightful comments and critical feedback provided have greatly enhanced both the scientific rigor and clarity of our work. Throughout the revision process, we have addressed each comment carefully and made substantial improvements to the manuscript, including expanded mechanistic discussions, enhanced methodological transparency, improved language quality, and the incorporation of additional analysis to reinforce key findings.

We affirm that the revised manuscript complies fully with all PLOS ONE editorial policies, ethical guidelines, and data availability standards. We have also ensured that all revisions are clearly marked and traceable, as required.

We sincerely value the opportunity to submit our work to PLOS ONE and hope that the revised manuscript now meets the expectations of the editorial team and reviewers. Should any further clarification or modification be needed, we remain fully committed to making additional improvements as guided by your expertise.

Thank you again for your time, thoughtful evaluation, and support throughout this process.

**Sincerely,**

Lixia Jiang

Heilongjiang Province Institute of Meteorological Science

Wuying National Climatological Observatory

hljjlx@163.com
